# CRISPR DNA elements controlling site-specific spacer integration and proper repeat length by a Type II CRISPR–Cas system

**DOI:** 10.1093/nar/gkz677

**Published:** 2019-08-08

**Authors:** Jenny G Kim, Sandra Garrett, Yunzhou Wei, Brenton R Graveley, Michael P Terns

**Affiliations:** 1 Department of Biochemistry and Molecular Biology, University of Georgia, Athens, GA 30602, USA; 2 Department of Genetics and Genome Sciences, Institute for Systems Genomics, University of Connecticut Health Center, Farmington, CT 06030, USA; 3 Department of Microbiology, University of Georgia, Athens, GA 30602, USA; 4 Department of Genetics, University of Georgia, Athens, GA 30602, USA

## Abstract

CRISPR–Cas systems provide heritable immunity against viruses by capturing short invader DNA sequences, termed spacers, and incorporating them into the CRISPR loci of the prokaryotic host genome. Here, we investigate DNA elements that control accurate spacer uptake in the type II-A CRISPR locus of *Streptococcus thermophilus*. We determined that purified Cas1 and Cas2 proteins catalyze spacer integration with high specificity for CRISPR repeat junctions. We show that 10 bp of the CRISPR leader sequence is critical for stimulating polarized integration preferentially at the repeat proximal to the leader. Spacer integration proceeds through a two-step transesterification reaction where the 3′ hydroxyl groups of the spacer target both repeat borders on opposite strands. The leader-proximal end of the repeat is preferentially targeted for the first site of integration through recognition of sequences spanning the leader-repeat junction. Subsequently, second-site integration at the leader-distal end of the repeat is specified by multiple determinants including a length-defining mechanism relying on a repeat element proximal to the second site of integration. Our results highlight the intrinsic ability of type II Cas1/Cas2 proteins to coordinate directional and site-specific spacer integration into the CRISPR locus to ensure precise duplication of the repeat required for CRISPR immunity.

## INTRODUCTION

CRISPR–Cas (Clustered regularly interspaced short palindromic repeats-CRISPR-associated) systems are diverse prokaryotic defense systems that provide immunity against viruses and plasmids (1,2). These adaptive immune systems are found in roughly half of bacteria and almost all archaea and fall into six distinct CRISPR–Cas types (I-VI) and over thirty subtypes that each utilize distinct components and mechanisms to achieve defense outcomes (3,4). CRISPR–Cas systems provide a heritable and sequence-specific method of protection against foreign invading elements by generating a memory of previous infections to elicit an effective immune response upon reinfection of the cell (5–8). Short invader-derived sequences are captured within the host CRISPR loci and used as templates to create short CRISPR RNAs that guide Cas proteins to recognize and cleave foreign genetic elements (9–15)

We are only now beginning to understand the detailed molecular mechanisms governing the capture of invader-derived sequences. This initial step in the CRISPR–Cas immune pathway is responsible for providing new, heritable immunity, and is referred to as ‘adaptation’. While details vary for the different types of CRISPR–Cas systems investigated thus far, adaptation generally involves the capture of foreign DNA and incorporation of that DNA into the host CRISPR locus, where the DNA fragments is then referred to as a ‘spacer’. The spacer sequence acts as a memory of the corresponding sequence within the foreign genome (called the protospacer). The foreign DNA must undergo processing steps prior to integration, during which time it is referred to as a pre-spacer (2,16). The CRISPR locus consists of a variably-sized leader sequence flanking an array of repeats separated by the similarly sized, previously incorporated spacers. The leader sequence harbors promoter elements used for crRNA expression as well as elements that guide the integration of new spacers at the leader-proximal repeat. (8,17–20). After addition of a new spacer, it has been shown that DNA repair machinery fills in DNA gaps and ensures faithful duplication of the CRISPR repeat such that a full repeat–spacer unit is added to the CRISPR locus (6,21) and Supplementary Figure S1). It is essential that both the sequence and length of the new repeat be preserved since the CRISPR repeats function both at the RNA level in crRNA biogenesis/function (18,22,23) and at the DNA level as they are the recipient site for addition of new spacers at the CRISPR locus. New spacers are added to CRISPR arrays in a polarized manner with the vast majority of spacers being incorporated at the leader-proximal repeat rather than downstream repeats (5,6,21,24). CRISPR-captured spacers located adjacent to the leader are often more highly expressed and more efficient in mediating interference against the invading nucleic acid than spacers present near the trailer end of the CRISPR array (25,26).

While several proteins have been shown to participate in the adaptation process in the various types of CRISPR systems (27–41), Cas1 and Cas2 are core components required for spacer integration *in vivo* and *in vitro* in nearly all CRISPR systems examined to date (3). Cas1 functions as the integrase that catalyzes spacer integration into the CRISPR repeat while Cas2 appears to primarily serve a structural role in the formation of a stable Cas1–Cas2 integrase complex (42–49). In both type I and type II systems, structural studies revealed that the integrase complex consists of a Cas2 dimer sandwiched between two Cas1 dimers; the complex binds pre-spacer DNA substrates and catalyzes integration of the two pre-spacer ends into the borders of a CRISPR repeat (44,46–50). Although Cas1 and Cas2 are the most highly conserved Cas proteins, *cas1* and *cas2* gene sequences vary, and *cas1* gene variability is one important basis for classifying CRISPR systems into types and subtypes (3). Sequence differences between Cas1 and Cas2 proteins likely underlie observed functional variability observed for integration in distinct CRISPR–Cas systems.

Recent *in vitro* studies with Cas1 and Cas2 from various type I and type II systems have provided key insight into the mechanisms of spacer integration into CRISPR repeats. The Cas1 and Cas2 complex can catalyze integration of the two ends of the pre-spacer independently, with a single integrated end referred to as a half-site event (17,42,48,49). *In vitro*, half-site integrations of spacer DNA can either proceed to full-site integrations (which results in complete insertion of spacer DNA into a CRISPR repeat) or they can be reversed by Cas1-mediated disintegration (7,42,51). Productive, full-site pre-spacer integration requires two concerted transesterification reactions in which the 3′-OH groups of the pre-spacer DNA carry out nucleophilic attacks at the 5′ ends of the repeat borders (17,42,49,50) and Supplementary Figure S1). Whether the two nucleophilic attacks required for full-site integration proceed with a set directionality or not for a given system is the subject of ongoing investigation. Recent studies suggest that type I and II systems first attack the top strand at the leader-repeat junction (LR), followed by a second attack of the repeat–spacer junction (RS) on the bottom strand (5,24).

Both leader and repeat sequences are relatively conserved among related CRISPR–Cas systems and *in vivo* and *in vitro* mutational analyses have provided evidence for a role of leader and repeat elements in specifying accurate integration of spacer DNAs into CRISPR arrays (7,20,26,42,49,52). Depending upon the system under investigation, polarized addition of spacers at the leader-proximal repeat can be mediated either by a protein factor, illustrated by the requirement for integration host factor (IHF) in type I-E and I-F systems (28,38,48,53,54) or by intrinsic properties of the Cas1–Cas2 integrase complex (7,49). Specific elements within the repeats have been shown to govern pre-spacer integration, but motifs vary for the types of repeats investigated (7,8,17,20,42,49,52,55–57). Of note, there is *in vitro* evidence from type I-E (56) and I-B (52) systems for key regions within the repeat that serve as ‘molecular rulers’ to guide integration to a defined distance away from these elements. It is unknown what mechanisms other CRISPR systems (e.g. type II) employ to ensure accurate integration and to maintain repeat length within a CRISPR array.

The seminal discovery that CRISPR–Cas systems function as adaptive immune systems was made following phage infections of the bacterium, *Streptococcus thermophilus* (1). *Streptococcus thermophilus* strain DGCC7710 remains one of the few organisms shown to incorporate spacer DNAs from invading viral (phage) or plasmid DNAs under laboratory conditions and without a need to overexpress adaptation proteins (1,41,58). We and others have focused attention on determining the detailed mechanism of adaptation in this organism which harbors four distinct CRISPR–Cas systems (two type II-A systems, a type III-A system and a I-E system). Both type II-A systems (CRISPR1 and CRISPR3) are active in the adaptation process (59–61). Our *in vivo* genetic analyses of the CRISPR1 system revealed that robust spacer acquisition requires Csn2 and Cas9 in addition to Cas1 and Cas2. However, it was not clear whether Csn2 and Cas9 were influencing upstream steps such as protospacer selection and processing, downstream integration, or both. (29,41). Recent *in vitro* studies with the related type II-A system in *S. pyogenes* found that Cas1 and Cas2 are sufficient for spacer integration and that Csn2 and Cas9 likely play a role in an upstream process of protospacer generation rather than being directly involved in spacer integration into CRISPR loci (7).

In *S. thermophilus*, our *in vivo* mutagenesis experiments revealed that the repeat-proximal 10 bp of the leader were necessary and sufficient to guide integration of spacers to the leader-proximal repeat (20). Moreover, mutations at the leader-repeat junction disrupted adaptation while mutations at the repeat–spacer junction were tolerated (20). A detailed mutational analyses of the *S. thermophilus* CRISPR repeat has not yet been performed to understand the role that repeat sequences play in specifying high fidelity integration of spacer DNA precisely at the repeat borders. Supplementary Figure S1 displays a provisional model of *S. thermophilus* adaptation based on *in vivo* experiments conducted with *S. thermophilus* (20) and *in vitro* experiments with type I and II systems (7,44,50). To gain a more in-depth understanding of the mechanisms governing *S. thermophilus* type II-A CRISPR spacer acquisition, we reconstituted and characterized the pre-spacer integration reaction *in vitro*. Our results show that Cas1 and Cas2, likely functioning as a Cas1–Cas2 integrase complex, have an intrinsic ability to recognize sequences to catalyze pre-spacer integration with high specificity for the identical repeat junction utilized *in vivo* (20). The spacer integration reaction is a two-step process and proceeds in a directional manner whereby integration of spacer DNA at the leader-repeat junction precedes integration at the repeat–spacer junction. Our findings indicate that each integration relies on the recognition of distinct elements within the leader or repeat of the CRISPR array. Our results underscore the intrinsic capacity of *S. thermophilus* Cas1 and Cas2 to coordinate specific and directional spacer integration during the adaptation stage of CRISPR–Cas immunity.

## MATERIALS AND METHODS

### Plasmid construction

The leader sequence and two repeat–spacer units of the CRISPR array was PCR-amplified from the *S. thermophilus* genome and cloned into the pWAR228 backbone plasmid by overlap PCR to generate pCRISPR. Leader sequence mutations were generated via inverse PCR and ligation of linearized plasmid using pCRISPR as the template. All plasmid constructs were verified by DNA sequencing and are listed in Supplemental Table S1.

### Protein purification

The *cas1, csn2* and *cas9* genes were amplified by PCR from the *S. thermophilus* genome and cloned into pET expression vectors to generate 6x-histidine-tagged proteins at the C-terminus (pET21d;Cas1 and Cas9) or N-terminus (pET24d; Csn2). The *cas2* gene was subcloned into pBAT4 expression vector to generate 6x-histidine-tagged SUMO Cas2 proteins at the N-terminus (pSAT1 and pSENP kindly provided by Dr Scott Bailey, Johns Hopkins University). Expression vectors were transformed into *Escherichia coli* BL21-Star cells (DE3, Stratagene). Cells were grown at 37°C in 1 L cultures of Luria broth to an OD_600_ of 0.6, and protein expression was induced overnight at room temperature by the addition of ispopropylthio-β-d-galactoside (IPTG) to a final concentration of 1 mM. The cells were pelleted, resuspended in lysis buffer (20 mM Tris, 500 mM NaCl, 10% glycerol, 20 mM imidazole and 5 mM 2-mercaptoethanol (BME), pH 7.5) and disrupted by sonication (Misonix Sonicator 3000). The lysate was cleared by centrifugation at 3500 rpm for 20 min at 4°C and His-tagged proteins were purified by Ni^2+^ affinity column chromatography (1.5 ml of HisPur Ni-NTA Resin (Thermo Scientific)) using a stepwise increase of imidazole (20, 50, 100 and 500 mM). The protein samples were dialyzed at 4°C in dialysis buffer (20 mM Tris, 150 mM KCl, 10% glycerol, 5 mM 2-mercaptoethanol (BME), pH 7.5) prior to performing activity assays. Purified proteins were analyzed by SDS-PAGE followed by Coomassie blue staining (Supplemental Figure S2).

### Generation of DNA substrates

DNA oligonucleotides were from Eurofins MWG Operon with the exception of hairpin DNA substrates used in Figure 4, which were from Integrated DNA Technologies and the sequences are given in Supplemental Table S2. Oligonucleotides were annealed by an incubation temperature gradient for 1 min at 95°C decreasing by 1°C each minute, down to 23°C. Annealed double-stranded substrates were run on a non-denaturing 15% polyacrylamide gel containing 1× TBE (89 mM Tris base, 89 mM Boric acid, 2 mM EDTA, pH 8.0), followed by ethidium bromide post-staining to verify proper annealing prior to radiolabeling. The annealed DNA substrates used as pre-spacers were 5′ end-labeled with T4 polynucleotide kinase (New England Biolabs (NEB)) in a 20 μl reaction containing 20 pmol oligonucleotide, 150 μCi of [γ-^32^P] ATP (6000 Ci/mmol; Perkin Elmer), 1× T4 PNK buffer and 10 U of T4 kinase (NEB).

### Integration assay with radiolabeled pre-spacer

For plasmid integration assays, individually purified recombinant Cas1 and Cas2 proteins at 2.5 μM each were added to a reaction containing 5 nM plasmid DNA, 20 nM 5′[γ-^32^P] ATP-radiolabeled DNA pre-spacer substrate, and integration buffer (20 mM Tris (pH 7.5), 100 mM KCl, 10 mM MnCl_2_, 5 mM 2-mercaptoethanol). This reaction was incubated at 37°C for 1 h and then quenched by the addition of 1 μg Proteinase K (ThermoFisher Scientific), 0.5% SDS, 1 mM EDTA and incubated at 50°C for 30 min. The products were analyzed on a 0.8% agarose gel pre-stained with ethidium bromide. After gel electrophoresis, the gels were dried on blot absorbent filter paper (Bio-Rad) overnight at room temperature using a vacuum gel dryer (Bio-Rad, Model 583 Gel Dryer). Radioactivity was detected with a phosphorimager (Storm 840 Scanner GE Healthcare).

For linear DNA target integration assays, individually purified recombinant Cas1 and Cas2 proteins both at 250 nM were added to a reaction containing 100 nM DNA CRISPR target, and integration buffer (described above). This reaction was incubated at 25°C for 5 min and then 20 nM 5′[γ-^32^P] ATP-radiolabeled DNA pre-spacer substrates were added and 10 μl samples were removed at 15 sec, 1 min and 15 min or incubated at 25°C for 1 hour. Reactions were quenched by the addition of equal volume (10 μl) of 95% formamide and 50 uM EDTA and incubated at 98°C for 5 min and separated on a 12% (8.0 M urea) denaturing polyacrylamide gel. Radiolabeled Decade Markers (Life Technologies) were used to determine the size of observed products. After gel electrophoresis, the gels were dried for 1 h at 90°C (Bio-Rad, Model 583 Gel Dryer) and radioactivity was detected by phosphorimaging as described above.

### Repeat mutation adaptation assay *in vivo*

For *in vivo* integration assays, pCas1/Cas2/Csn2/Cas9 with a minimal CRISPR array (pCRISPR) was used as template as previously described (41) and inverse PCR was used to introduce both insertions and deletions of the repeat sequence. Plasmid constructions were verified by sequencing and transformed into *S. thermophilus* DGCC7710 strain via electroporation (59). *S. thermophilus* harboring the plasmids were grown in LM17 liquid medium supplemented with 2 μg/mL chloramphenicol for 16 hours. Cells from each strain were harvested, pelleted and genomic DNA was extracted using the Zymo Research Quick-DNA Fungal/Bacterial Miniprep Kit (Zymo Research, Irvine CA) and used as PCR template. Primers matching the leader and plasmid sequence were used for PCR amplification of the CRISPR array on the plasmid. PCR products were run on 2.5% TAE-agarose gels, pre-stained with ethidium bromide to assess CRISPR array expansion. Bands representing an expanded CRISPR array were gel excised using the Zymo Gel Extraction DNA Recovery Kit (Zymo Research, Irvine, CA, USA), purified and sequenced by high-throughput sequencing. Plasmid constructs are listed in Supplemental Table S1.

### Pre-spacer integration high-throughput sequencing

#### Library preparation

To sequence integration events, the spacer integration assay was performed as described above using unlabeled pre-spacer. After incubation, DNA was isolated using the DNA Clean and Concentrator Kit (Zymo Research, Irvine, CA, USA). For the plasmid integration samples, excess un-integrated pre-spacer was removed using Agencourt AMPure XP beads (Beckman Coulter, Indianapolis, IN). Illumina adapter sequence with an N_10_ random primer was annealed to the plasmid DNA and extended (thermocycler conditions: 98°C for 30 s, 25°C for 30 s, 35°C for 30 s, 45°C for 30 s and 72°C for 5 min). Excess adapter was then removed using AMPure beads, and PCR was performed to amplify plasmid DNA that contained integrated pre-spacer: forward primers were specific for the pre-spacer, while reverse primers targeted the Illumina adapter introduced with the random anneal and extension step. The resulting amplicons captured both full-site and half-site integration events with no apparent discrimination. Illumina barcodes and adapter sequences were added with a final PCR and the resulting library was separated on a 1% agarose gel. DNA in a 400–700 bp size range was selected and isolated using the Zymo Gel DNA Recovery Kit (Zymo Research, Irvine, CA, USA). Sequencing was performed on an Illumina MiSeq with a 100 × 50 cycle run. Only the 100 bp Read 1 data was used in this analysis.

For the minimal linear CRISPR substrate products, 1 μl of eluted DNA was used as a PCR template. Primers to add Illumina adaptor sequences were annealed to the newly integrated spacer and the 3′ end of either the plus or minus strand of the CRISPR substrate. DNA Clean and Concentrator Kit (Zymo Research, Irvine, CA, USA) was used to isolate the PCR product, and 1 μl of this product was used as the template for a second PCR using primers to add Illumina barcodes. These products were purified on a 1% agarose gel and extracted with a Gel Purification Kit (Zymo Research, Irvine, CA, USA).

#### Mapping integration events

After sequencing, samples were de-multiplexed by barcode and analyzed to determine sites of integration. For plasmid data, the complete pre-spacer sequence was located in each read and 50 bp of sequence immediately downstream from the end of the pre-spacer was extracted and aligned to the appropriate plasmid reference using Bowtie (62). To visualize the distribution of integration events, alignment output files were converted into coverage files using bedtools (63) and displayed on a custom UCSC genome browser track hub (https://www.genome.ucsc.edu). To determine sequence preferences at the sites of integration, the base at the integration point, along with upstream and downstream context sequence, was extracted from the reference sequence with bedtools and used to make sequence logos (64). For the minimal linear CRISPR integration data, the spacer-target junction was determined from each read and counts for each potential integration point were totaled. Integration events are displayed as the percent of total reads for each position along the CRISPR target.

#### Characterizing in vivo spacer integration into pCRISPR with repeat mutations

Size selected and purified array amplicon libraries were sequenced on an Illumina MiSeq with a 250 × 50 cycle run (250 bp Read 1 data used in this study). Samples were de-multiplexed by barcode and then analyzed with custom python scripts to determine how new spacers were integrated. Briefly, the leader-repeat junction and the beginning of the second repeat were located in each read. The beginning of the second repeat was defined as the 3′ end of a set of hypothetical spacers, which ranged in size from 27 to 33 bp. This size range captures 99.9% of new type II-A spacers observed in spacer uptake assays with wildtype *S. thermophilus*. Each of the seven hypothetical spacers was aligned to a reference sequence including the genome and plasmid sequences using bowtie (62). Alignment outputs were then examined to determine the longest hypothetical spacer that aligned with no mismatches. This hypothetical spacer was considered the ‘true’ new spacer and its length was used to locate the position of the repeat–spacer junction, thereby allowing us to identify the integration site for each read. The number of reads supporting integration at each position along the pCRISPR array was counted and summarized and events are displayed as the percent of total reads for each position along the pCRISPR array.

## RESULTS

### *S. thermophilus* Cas1 and Cas2 accurately integrate spacer DNA at the leader-proximal repeat *in vitro*

**Figure 1.**
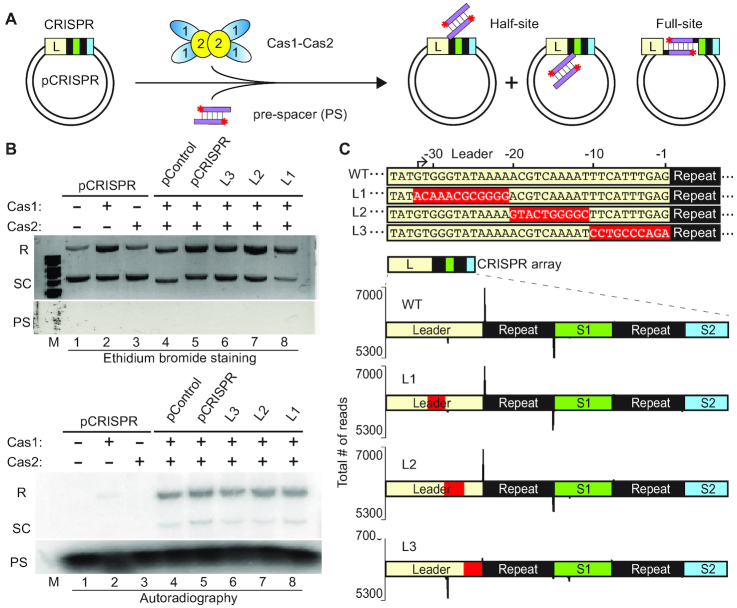
*S. thermophilus* Cas1 and Cas2 accurately integrates pre-spacers *in vitro* and 10 bp of the leader sequence is essential for polarized integration. (**A**) Schematic of pre-spacer (PS) integration by Cas1–Cas2 into a plasmid target containing a minimal CRISPR array (pCRISPR). Integration of pre-spacers can occur as half-site intermediates at either junction of the repeat or as full-site products. (**B**) Integration assays with Cas1–Cas2 and radiolabeled pre-spacers visualized with ethidium bromide staining and autoradiography. Integration products corresponding to relaxed plasmids (R), unintegrated supercoiled plasmid (SC) and free pre-spacers (PS) are indicated. (**C**) Variants of the leader sequence mutations (L1, L2, L3) engineered on pCRISPR. Sites of spacer integration were identified by high-throughput sequencing and mapped to the plasmids on the plus (upper) and minus (lower) strands.

To investigate mechanisms directing spacer DNA uptake into CRISPR loci by the *S. thermophilus* type II-A CRISPR–Cas system, we established an *in vitro* system capable of accurately integrating pre-spacer (PS) donor DNA substrates into CRISPR DNA recipient molecules (Figure 1). Purified recombinant *S. thermophilus* Cas1 and Cas2 (Supplementary Figure S2) were incubated with 5′-radiolabeled double-stranded DNA pre-spacers with 5 nt 3′-overhangs and a plasmid (pCRISPR) containing a minimal CRISPR array consisting of the full, 157 bp leader and two repeat–spacer units (Figure 1A). The leader used in pCRISPR was either wildtype or contained blocks of transition mutations upstream of the first repeat (–32 to –21 bp (L1), –20 to –11 bp (L2) and –10 to –1 (L3); Figure 1C). The pre-spacer design was based on a 30 bp substrate originating from the frequently acquired S4 sequence of the 2972 lytic phage (58), with overhangs to mimic a processed pre-spacer prior to integration. Consistent with type II-A (7) and type I-E (45) *in vitro* spacer integration assays, blunt-ended 30 bp substrates resulted in a less efficient spacer integration reaction compared to 3′-overhang substrates (data not shown). A plasmid lacking a CRISPR array (pControl) was used to observe any off-target spacer integration events. Spacer integration, as evidenced by incorporation of radiolabeled pre-spacer DNA into the recipient plasmid substrates, was observed for pCRISPR, all mutant leader variants of pCRISPR as well as pControl (devoid of a CRISPR array) (Figure 1B, lower panel, lanes 4–8). The formation of integration products was also deduced from changes in plasmid conformation: strand nicking during either half-site or full-site spacer integration (Figure 1A) converts the supercoiled (SC) plasmid into relaxed (R) forms (Figure 1B, upper panel, lanes 1–8). As expected, the majority of the radiolabeled integration products co-migrated with the relaxed form of the plasmid but a minor signal is observed at the position of the supercoiled form and likely reflects integration prior to relaxation of the supercoiled plasmid DNA (Figure 1B, lower panel, lanes 4–8). Both Cas1 and Cas2 were necessary for efficient integration, although very low levels of integration were reproducibly observed with Cas1 alone (Figure 1B, lower panel, compare lane 2 with lane 5) as has been observed in other *in vitro* integration studies (38,42,50).

The precise sites of all spacer integrations for each of the tested plasmids were determined by high-throughput DNA sequencing (Figure 1C and Supplementary Figure S3). Specifically, we used primers targeting the pre-spacer to make strand-specific amplicon libraries. Integration into pCRISPR occurred with high specificity for the first (leader-proximal) repeat, occurring at the same top strand and bottom strand repeat junctions as is observed *in vivo* (Figure 1C (WT)) (20). Upstream leader mutations did not disrupt this specificity (L1 and L2), but integration at the leader proximal repeat was dramatically impaired when the repeat-proximal 10 bp of the leader was mutated (L3) (Figure 1C; Supplementary Figure S3), revealing that this region of the leader is critical for guiding integration to the appropriate leader-adjacent repeat. These results show that Cas1 and Cas2 are sufficient to faithfully recapitulate spacer integration at the leader-adjacent repeat of a CRISPR array as is observed *in vivo* and that the adjacent 10 bp of the leader region is critical for guiding integration to the appropriate repeat.

**Figure 2.**
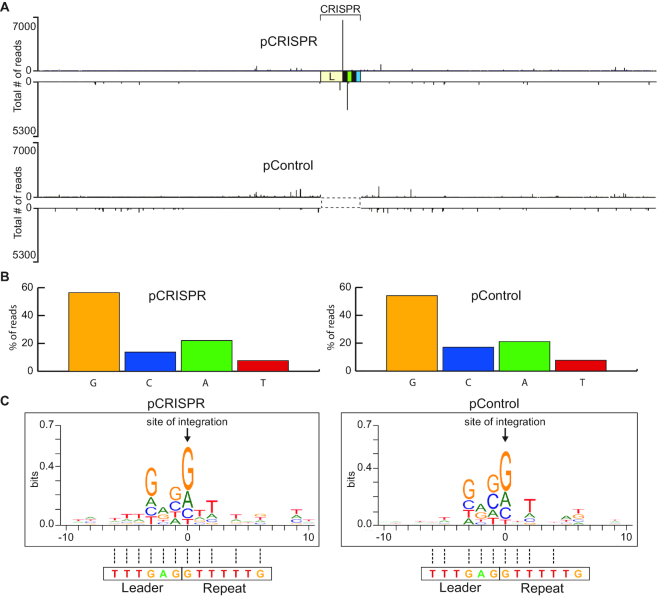
Non-CRISPR integration sites resemble a leader-repeat junction. (**A**) Sites of spacer integration for pCRISPR and pControl were identified by high-throughput sequencing. (**B**) Percent of spacer integration sites occurring at a guanine, cytosine, adenine and thymine on pCRISPR and pControl. (**C**) WebLogo of all non-CRISPR integration sequences. Sequence homology to the actual leader-repeat junction sequence is indicated with dotted lines.

In addition to specific integration at the leader-proximal CRISPR repeat, we also observed low levels of integration at non-CRISPR sites that were broadly distributed throughout the plasmid backbone in both pCRISPR (containing a CRISPR array) and pControl (lacking a CRISPR array) (Figure 2A and Supplementary Figure S3). Analyses of these off-target sites, which likely represent half-site integrations, revealed a strong preference for guanine which is in agreement with the nucleotide identity of the natural *S. thermophilus* CRISPR repeat borders (Figure 2B). The base preference of integration was guanine (pCRISPR: 56.5%; pControl: 54.1%) followed by adenine (pCRISPR: 22.1%; pControl: 21.1%) and then cytosine (pCRISPR: 13.7%; pControl: 17.1%) and thymine (pCRISPR: 7.7%; pControl: 7.7%) (Figure 2B). In addition, there is an apparent preferred upstream and downstream sequence context for off-target integrations (Figure 2C) that resembles a leader-repeat junction sequence, further supporting an intrinsic sequence recognition by Cas1–Cas2.

### *S. thermophilus* Cas1–Cas2 integrates pre-spacers into linear CRISPR targets

**Figure 3.**
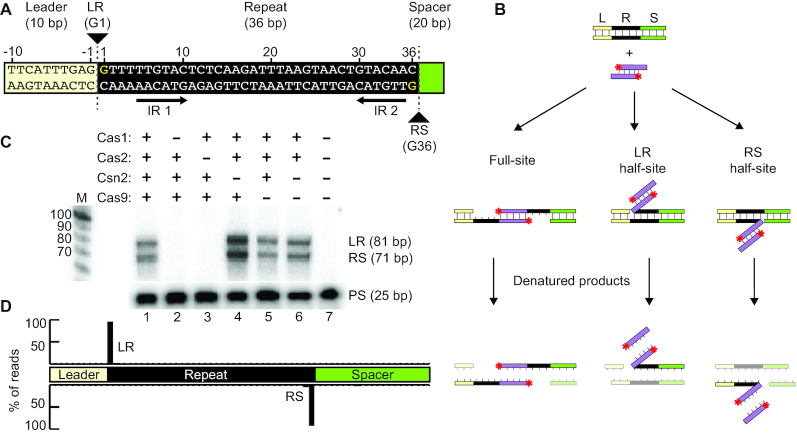
Specific pre-spacer integration by Cas1–Cas2 into linear dsDNA targets. (**A**) Representation of the linear CRISPR target. Target consists of a leader sequence (yellow), repeat (black) and spacer (green). Leader-repeat (LR) and repeat–spacer junctions (RS) and points of integration (G1 and G36) are indicated. Palindromic inverted repeat sequences (IR 1 and IR 2) are marked. (**B**) Schematic of spacer integration *in vitro* with a minimal linear CRISPR target. Integration products include full-site integration, or half-site integrations at either the leader-repeat junction (LR) or repeat–spacer junction (RS). (**C**) *In vitro* spacer-integration assay with Cas1, Cas2, Csn2 and/or Cas9. Expected integration products corresponding to both junctions (LR or RS) are indicated. (**D**) High-throughput sequencing analysis of integration products represented as percent of total reads mapped throughout the linear CRISPR target.

The plasmid integration assay described above (Figure 1) demonstrated that *S. thermophilus* Cas1 and Cas2 show high specificity for integrating spacers at the leader-proximal repeat, but it did not allow us to distinguish half-site vs. full-site spacer integration or reveal the potential order of the two nucleophilic attacks. To address these questions, we employed a minimal linear CRISPR target consisting of 10 bp of the leader sequence, a single 36 bp repeat, and a single 20 bp spacer (Figure 3A). We observed specific integration of radiolabeled pre-spacers at the repeat borders of this linear CRISPR target as evidenced by bands of the expected sizes for spacer integration at the LR and RS junctions (Figure 3B and C). The sites of integration at the LR and RS borders were also analyzed by high-throughput sequencing, again using a strand-specific amplicon approach. Sequencing reads revealed that integration occurred precisely at the first and last nucleotides of the repeat (Figure 3D). We previously found that four proteins (Cas1, Cas2, Csn2 and Cas9) are required for new spacer addition to CRISPR arrays *in vivo* (41). We tested the importance of each protein for carrying out *in vitro* spacer integration (Figure 3C and purified proteins shown Supplementary Figure S2) and we found that Cas1 and Cas2 proteins are sufficient for specific integration (Figure 3C, lane 6). The integration levels observed with Cas1 and Cas2 appeared to be unaffected by addition of Csn2 (lane 5) and slightly enhanced by Cas9 (lane 4), through an unknown mechanism. High-throughput sequencing of the integration reaction products showed that Cas1 and Cas2 alone are capable of highly specific integration at the repeat borders with at least 92% of all pre-spacers mapping to the LR and RS repeat junctions (Figure 3D). These results show that sequence specificity of the *S. thermophilus* Cas1–Cas2 integrase complex is sufficient for accurate integration into a minimal linear CRISPR target *in vitro*.

### Full-site integration of pre-spacers by Cas1–Cas2 is directional

**Figure 4.**
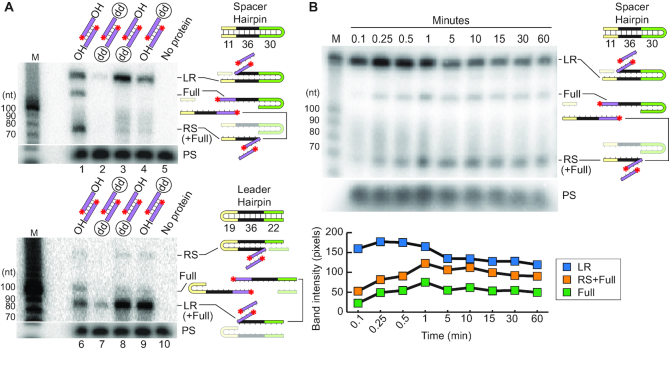
Cas1–Cas2 spacer integration reaction is directional. (**A**) Detection of full-site and half-site integration products with spacer hairpin CRISPR targets (top panel) and leader hairpin CRISPR targets (bottom panel) with unmodified (OH/OH) and modified (OH/dd, dd/OH, dd/dd) pre-spacers show site of the first transesterification reaction. (**B**) Time-course of spacer integration assay using unmodified pre-spacer and spacer hairpin CRISPR target. Quantification of B (bottom panel).

We next examined if *S. thermophilus* Cas1–Cas2 was capable of catalyzing full-site and accurate spacer integrations and if there was a preference for first site integration at the leader-repeat or repeat–spacer junction (Figure 4). These experiments were conducted using CRISPR targets with a DNA hairpin structure at either the spacer end or leader end to enable full-site products to be distinguished from half-site products on the basis of size (Figure 4A and B). In addition, we compared integration patterns for pre-spacers having natural 3′ hydroxyl end groups (capable of executing two nucleophilic attacks for full-site integration) with those containing a single 3′ dideoxy (dd) group on one strand or the other (can undergo just one site of integration) or having dideoxy groups at both ends (to block all 3′ hydroxyl-catalyzed-mediate integrations). Pre-spacers with 3′-OH termini underwent full-site integration at both LR and RS borders (Figure 4A). In contrast, for pre-spacers with a single modified dideoxy terminus, the majority of the integration products were leader-repeat half-site intermediates for both spacer and leader hairpin targets (Figure 4A, lanes 3, 4, 8 and 9) indicating that the first nucleophilic attack occurs at the leader-repeat junction rather than the repeat–spacer junction. As expected, integrations were blocked when the dideoxy was present on both strands of the pre-spacer (the low background of observable integration is likely due to lack of dideoxy groups on a small fraction of the pre-spacer substrates or a less efficient nucleophile in the reaction (e.g. H_2_O)) (Figure 4A, lanes 2 and 7).

A time course analysis of the integration reaction with pre-spacers with 3′-OH termini provided additional evidence that the leader-repeat junction is preferentially recognized vs. the repeat–spacer junction (Figure 4B). Within 6 s of initiating the reaction, leader-repeat half-site intermediates were the most abundant product and accumulated prior to the appearance of repeat–spacer half-site intermediates and full-site (LR + RS) integration products (Figure 4B, top panel). Quantification of the half-site integration intermediates and full-site products with time showed that leader-repeat half-site intermediates are the most abundant products throughout the reaction and that progression to full-site integration correlated with a steady decrease in leader-repeat half-site integrations (Figure 4B, bottom panel). Together, these results show that full-site spacer integration reactions facilitated by *S. thermophilus* Cas1–Cas2 proceed in a sequential manner with the first reaction occurring at the leader-repeat junction followed by a subsequent second integration at the repeat–spacer junction.

### Important elements of the CRISPR repeat

**Figure 5.**
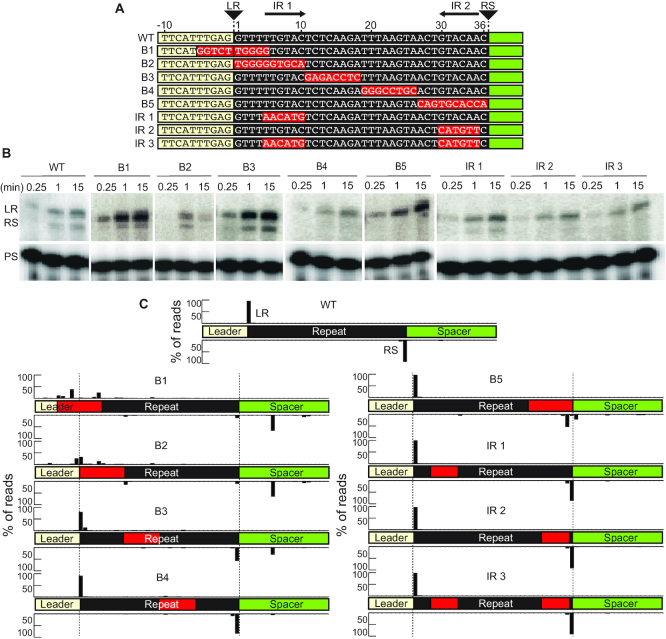
Repeat sequence mutations affect efficiency and specificity during spacer integration. (**A**) Annotation of leader and repeat sequence mutations on the linear CRISPR target. Leader-repeat junction (LR), Repeat-spacer junction (RS) and inverted repeats (IR1 and IR2) are indicated. (**B**) Integration reaction with mutated CRISPR targets taken at time points: 15 s, 1 min and 15 min. (**C**) High-throughput sequencing analysis of strand-specific integration products; peaks represent the percent of total reads mapped throughout the linear CRISPR target. Top strand and bottom strand libraries were prepared separately and read counts are normalized across strands. Red boxes indicate mutated sequences in the CRISPR target. Nucleotide level resolution of high-throughput sequencing data is provided in Supplemental Figure S4. Range of total number of reads (4,711–12,359).

Having determined the important role of the first 10 bp of the leader sequence in directing spacer integrations at the leader-proximal repeat (Figure 1), we next investigated sequence determinants within the repeat important for guiding integration by the Cas1–Cas2 complex (Figure 5). We introduced a series of block substitution mutants to a minimal linear CRISPR target (Figure 5A) and evaluated the effects of each mutation relative to the wildtype repeat, on spacer integration efficiency (level of integration products observed by gel separation and autoradiography; Figure 5B) and specificity (location of integration determined and quantified through strand-specific sequencing; Figure 5C and see Supplementary Figure S4 for detailed mapping of integration sites). Mutation of sequences spanning the leader-repeat junction (mutant B1) abolished spacer integration at both LR and RS junctions with only a relatively moderate reduction in overall integration efficiency. Likewise, mutation of leader-proximal region of the repeat (mutant B2) also resulted in a similar loss of specificity at both junctions of the repeat and significantly impaired the efficiency of integration. Mutation of a mid-repeat sequence block towards the leader (mutant B3) did not significantly impact integration specificity or integration efficiency. However, mid-repeat sequence mutations towards the spacer end of the repeat (mutant B4) as well as for mutations in one (mutants IR 1 and IR 2) or both (mutant IR 3) of the palindromic repeats did not significantly impact integration specificity, despite leading to a significant reduction in the efficiency of spacer integration at the second site of the repeat–spacer junction. Mutation of a sequence block adjacent to the repeat–spacer border (mutant B5) resulted in a loss of specificity at the second site of the repeat–spacer junction but not the first (LR) site of integration while efficiency at the second site was significantly reduced. We note that mutations that affect specificity of the first site of integration at the LR junction (e.g. mutants B1, B2 and to a lesser extent B3) also resulted in a loss of specificity at the second site of integration at the RS border. The inverse was not true as illustrated by the block 5 mutant (B5) which is capable of integration at the LR but not RS junctions. Furthermore, we observed a relatively prominent aberrant integration eight bases downstream of the RS border at a guanine in the spacer region occurring for both B1 and B2 (and to a reduced degree with B3) mutants that is not observed with the WT repeat (Figure 5C and Supplementary Figure S4). Together, the results support a role for several repeat sequences in determining the efficiency and/or specificity of integrations by Cas1–Cas2 and also provide support for a two-step integration reaction whereby accurate first step integration is a prerequisite for achieving accurate full-site integration.

**Figure 6.**
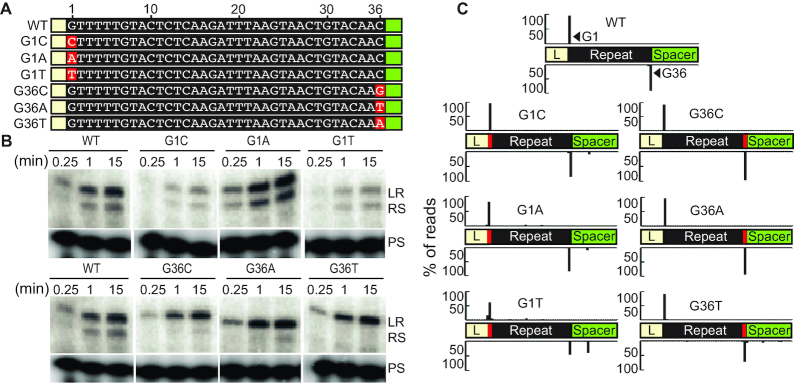
Identity of the first and last nucleotide of the repeat affect efficiency and specificity during spacer integration. (**A**) Annotation of repeat sequence mutation of guanine at position 1 and position 36. (**B**) Integration reaction with mutated CRISPR targets taken at time points: 15 s, 1 min and 15 min. (**C**) High-throughput sequencing analysis of integration products represented as percent of total reads mapped throughout the linear CRISPR target. Guanine at position 1 and 36 of the repeat are displayed as G1 and G36. Nucleotide level resolution of high-throughput sequencing data is provided in Supplemental Figure S4. Range of total number of reads (6,114–16,093).

Next, to understand if the identity of the two nucleotides which serve as the sites of nucleophilic attack during spacer integration (G1 on the top strand and G36 on the bottom strand), is important for directing integration by Cas1–Cas2, we assayed each possible combination of nucleotides at these two positions (Figure 6 and see Supplementary Figure S4 for detailed mapping of integration site). Mutation of the guanine in position 1 to a cytosine (G1C) or adenine (G1A) did not impact integration specificity at either LR or RS junction, while a moderate defect in specificity at both junctions was observed for the thymine substitution (G1T) at position 1 (Figure 6C) and this led also to aberrant integration within the spacer region at a guanine (Figure 6C and Supplementary Figure S4). A reduction in integration efficiency was observed for both the G1C and G1T mutations but not the G1A mutation (Figure 6B). At the last position of the repeat, mutation from a guanine to all other nucleotides (G36C, G36A and G36T) did not significantly affect integration specificity at either junction of the repeat or integration efficiency at the first site (LR border). However, all three changes to nucleotide 36 impaired efficiency of integration at the second site (RS border) (Figure 6B, C). Thus, the identity of the base at the sites of transesterification attack on the CRISPR repeat is an important component for specifying efficient and/or specific integration at a CRISPR repeat by the Cas1–Cas2 complex.

### Second-site integration is defined by a molecular ruler-based mechanism

**Figure 7.**
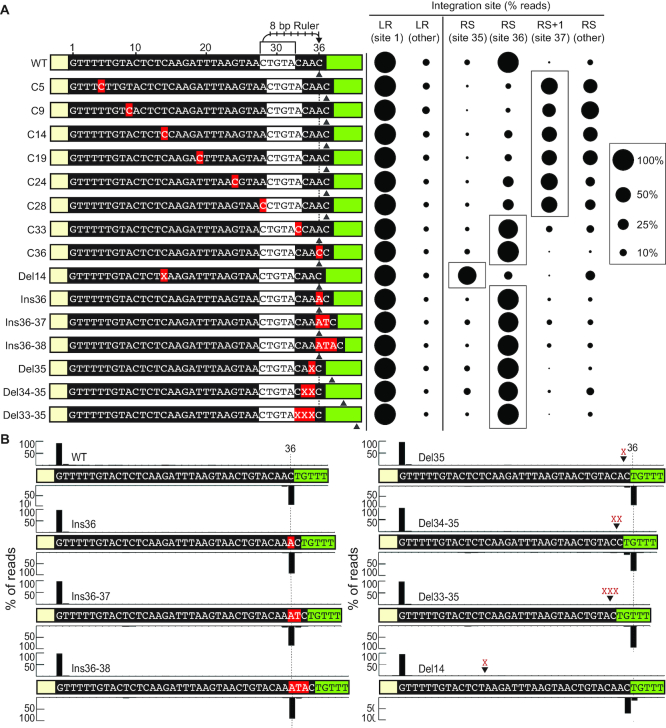
Second-site integration at the repeat–spacer junction is defined by a molecular ruler. (**A**) (Left panel) Single, double and triple nucleotide insertion and deletion mutations made to the minimal CRISPR target. Mutations in red boxes are indicated. Predicted 5′-CTGTA-3′ ruler element defining second-site transesterification attack 8 bp from the 5′-C is marked. Dotted line marks position 36 of the repeat. Grey arrow indicates the preferred site of integration for each CRISPR target. (Right panel) Sites of integration represented as percent of total mapped reads at the leader-repeat junction (LR) and positions spanning the repeat–spacer junction (RS) qualitatively represented (right panel). Nucleotide level resolution of high-throughput sequencing data is provided in Supplemental Figure S4. (**B**) Integration sites for single, double and triple insertion and deletion mutations mapped to the minimal CRISPR target at nucleotide resolution. Position 36 is indicated with a dotted line. Range of total number of reads (14,884–293,723).

Our findings support a model where full-site spacer integration proceeds in a directional manner such that integration at the leader-repeat junction (site 1) occurs prior to integration at the repeat–spacer junction (site 2) with the first integration being governed by sequence-specific interactions of Cas1–Cas2 and sequences spanning the leader-repeat junction. We next investigated how the second site of integration at the far end of the repeat (G36) is orchestrated (Figure 7). Similar to what has been observed for *in vivo* type I-E and I-B adaptation studies (52,56), we tested whether the site of the second integration would be directed by a region within the repeat that acts as a molecular ruler to determine the distance of the second nucleophilic attack in a sequence-independent fashion. In the type I studies, the repeat regions determined to act as molecular rulers were identified by testing the effects of strategically located nucleotide insertions or deletions within repeats on defining the site of the second step of integration. Altering the length of the repeat upstream of the ruler element shifted the second integration site upstream or downstream a fixed distance dictated by the length of the insertion or deletion. In contrast, insertions or deletions downstream of the ruler element resulted in second-site integrations occurring at a fixed short distance (typically 8–10 bp depending on the system) downstream of the motif to a common location (52,56). Accurate integration precisely at the two repeat borders is required to maintain repeat length which in turn is critical for generating a functional CRISPR array capable of producing active crRNAs as well as accepting spacers from new viral invaders.

To test the hypothesis that type II repeats harbor an element that serves as a molecular ruler defining the second site of integration (in this case G36), single cytosine residues were inserted at regular intervals across the 36 bp repeat and the sites of integration for each mutant were quantified to determine any effects on the choice of second site integration (Figure 7A and see Supplementary Figure S4 for detailed integration site mapping). None of the C insertions impacted accurate integration at the leader-repeat border (site 1 at G1). However, significant differences were observed in the location of the second site of integration (site 2) depending upon if the C were inserted before or after position 28 (Figure 7A). Specifically, we found that when C insertions were introduced at locations upstream of position 28 (mutants C5, C9, C14, C19, C24, C28), then second-site integration occurred one base further down (i.e. position 37) than WT repeat (position 36) and there were spurious sites not observed with WT repeats (see Supplementary Figure S4 for locations of all sites of integration). Moreover, deletion of a C upstream of position 28 (mutant Del14 at position 14) resulted in a shift in the second site of integration one base upstream (i.e. position 35) than WT repeat (position 36). In contrast, when the C insertions were performed at or downstream of position 33 (mutants C33, C36), integrations occurred at the same site as the WT repeat (position 36). Additionally, we found that the second site of integration remained at the 36th position of the repeat even when single, double or triple insertion (mutants Ins36, Ins36-37, Ins36-38) or deletions (mutants Del35, Del34–35, Del33–35) were introduced with low levels of aberrant integration (Figure 7A and B). Together, the results indicate that the upstream region of the repeat can tolerate changes in length, but downstream of the 28–32 region, insertions or deletions result in off-site integration at any nucleotide 8 base-pairs away from position 28 of the repeat.

### *In vivo* evidence that the second-site integration step is governed by a molecular ruler-based mechanism

**Figure 8.**
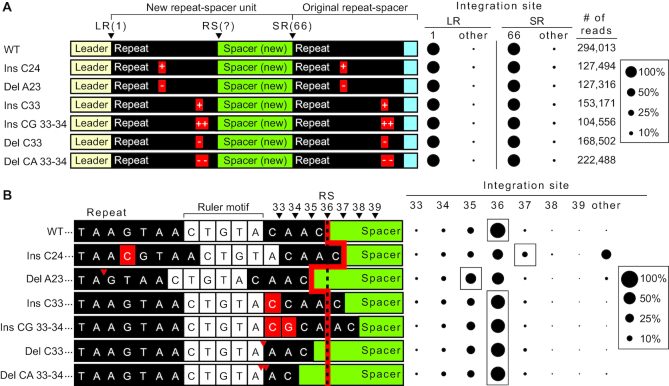
Ruler-based mechanism influences second-site integration *in vivo*. (**A**) (left panel) Annotated sequence of expanded CRISPR array *in vivo* with single and double insertion and deletion mutations in the repeat sequence. (Right panel) Sites of integration represented as percent total of mapped reads at the leader-repeat junction (LR) and spacer–repeat junction (SR). (**B**) (left panel) Sequences spanning the repeat–spacer junction and site of integration. Red line highlights expected site of integration relative to insertions and deletions upstream and downstream of the ruler element (5′-CTGTA-3′) as indicated by red arrows or boxes. (Right panel) Integration site at the positions spanning the repeat–spacer junction.

Finally, we tested if the ruler-based mechanism governing the site of the second step of *in vitro* integrations also operates *in vivo*. Similar to our *in vitro* mutational analysis (Figure 7), we introduced nucleotide insertions or deletions, both upstream and downstream of the ruler element located between position 28 and 32, into repeats on pCRISPR (Figure 8). Plasmids were then transformed into *S. thermophilus* cells and new spacer acquisition was determined using a PCR based approach (20) combined with high-throughput sequencing of the expanded CRISPR arrays. Expanded arrays were observed for all mutants, however the overall efficiency of spacer integration was often noticeably reduced (Supplementary Figure S5). None of the insertion or deletion mutations affected the accuracy of integration at the first leader-repeat (LR) border and the downstream spacer–repeat (SR) border was also preserved (Figure 8A). In contrast, differences in the second site of integration at the repeat–spacer (RS) border were observed for some of the repeat mutants (Figure 8A). Similar to what we observed *in vitro* (Figure 7), single nucleotide insertion (Ins C24) or deletion (Del A23) upstream of the ruler element resulted in a corresponding shift in the site of integration by one nucleotide downstream (position 37) or upstream (position 35) compared to WT (position 36), respectively. We note that integration at position 36 for these two mutants was also observed at a relatively high level compared to what was observed *in vitro*, indicating that compensatory mechanisms appear to operate *in vivo* to find the natural RS junction despite the introduced point mutations within the repeats. For the insertion mutant (Ins C24), we also observed significant aberrant second-site reactions (marked ‘other’ in Figure 8B) that mostly correlate to an attack within the spacer at position 45 which is the same guanine observed with our *in vitro* mutational results (eight bases downstream of the RS border in the spacer region, see Supplementary Figure S6B and see Supplementary Figure S4 for detailed integration site mapping). This improper second-site reaction results in partial duplication of the spacer during full-site integration, and is not observed in WT (Supplementary Figure S6A). As predicted for the molecular ruler model, insertions (Ins C33, Ins CG 33–34) or deletions (Del C33, Del CA 33–34) downstream of the ruler element did not affect site of integration at the second-site and maintained a preference similar to WT for position 36. Sequencing results showed that positioning was maintained by either the loss of 1 nucleotide from the 3′ end of the mutant repeat or the addition of 1 nucleotide corresponding to the first base of the previous spacer. Together, these results show that *in vivo*, second-site integration is influenced by a ruler-based distance mechanism.

## DISCUSSION

Successful acquisition and integration of new spacers into the CRISPR locus is a fundamental step for heritable CRISPR–Cas immunity against viruses and other potentially harmful or lethal mobile genetic elements. With each new spacer acquired, there is an accompanying duplication of the repeat due to DNA repair of the gapped DNA intermediate containing the integrated spacer flanked on either side by single-stranded repeat sequences (Supplementary Figure S1). When spacer integration occurs accurately at 5′ nucleotides that comprise the leader-repeat (LR) and repeat–spacer (RS) borders, DNA repair processes (polymerase fill-in and ligation reactions) yield a new repeat that is a perfect copy of the original repeat (8,30). Subsequently, the newly generated repeat at the leader end of the CRISPR array is competent to function as the recipient structure for subsequent addition of the next spacer. The periodicity of the repeat–spacer units of the entire CRISPR repeat is maintained even after multiple novel spacer additions. In addition to its role in permitting accurate array expansion, the repeat could also influence the biogenesis of functional crRNAs. Transcribed type II repeat sequences must match and bind tracrRNA, be processed by RNase III, and ultimately portions of the repeat RNA (referred to as 5′ or 3′ ‘tags’ or ‘handles’) are key elements of mature crRNAs and are critical for crRNA–Cas protein assembly and function in crRNA-guided invader nucleic acid destruction (11,12,65,66). Thus, imprecise full-site integration of spacers has the potential to lead to inactive CRISPR arrays and/or non-functional crRNAs.

Our work provides the first *in vitro* characterization for spacer integration for the type II-A CRISPR–Cas system of *Streptococcus thermophilus*. We established an *in vitro* system capable of accurately integrating full-site spacer DNA at the proper junctions of *S. thermophilus* CRISPR repeats and importantly, our characterization of the reaction revealed key mechanistic information for how Cas1 and Cas2 accurately integrate new spacers in a polarized manner at the leader-adjacent repeat. Our approach of analyzing *in vitro* integration products through gel electrophoresis to address efficiency, combined with sequencing to address integration site specificity, provided a more comprehensive approach to studying integration than previous studies that relied on either gel analysis or sequence analysis alone (7,49,52,56).

### Model for type II spacer integration at CRISPR arrays

**Figure 9.**
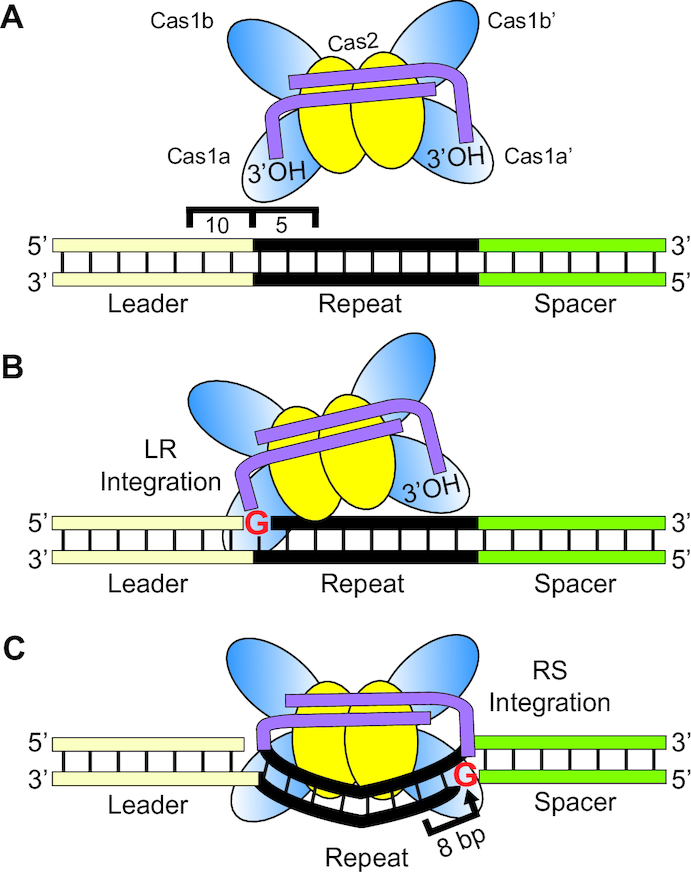
*S. thermophilus* type II-A spacer integration model. (**A**) The integrase complex (Cas1–Cas2 bound by a pre-spacer) recognizes 15 bp of the sequences spanning the leader-repeat junction to direct specific integration to the first repeat. (**B**) Identity of a guanine at position 1 of the repeat facilitates localization of integration to direct the first transesterification attack at the leader-repeat junction, resulting in a half-site intermediate. (**C**) DNA bending of the repeat sequence initiates the second-site transesterification attack at the repeat–spacer junction measuring 8 nucleotides upstream of a molecular ruler localized near the repeat–spacer junction (Figures 7 and 8). The integration complex additionally relies on a guanine at position 36 to progress to full-site integration of a new spacer into the CRISPR array.

Our results are consistent with a model (Figure 9) whereby *S. thermophilus* Cas1 and Cas2, likely functioning as a Cas1–Cas2 integrase complex that binds the spacer substrates (49), catalyze spacer integration specifically at the leader-proximal repeat through a two-step transesterification reaction. The 3′ hydroxyl groups of the DNA spacer each carry out nucleophilic attacks at the borders of the first repeat sequence, on opposite strands (Figure 9A–C). Several lines of evidence indicate that there is an apparent obligate order to the two nucleophilic attacks whereby the first attack occurs on the top strand at the guanine of the leader-repeat junction (LR) and the second attack is made at the guanine of the repeat–spacer junction (RS) on the bottom strand (Figure 9B and C). For example, integration occurred selectively at the LR rather than at the RS junction when pre-spacers had only a single unmodified dideoxy terminus available for nucleophilic attack. (Figure 4). Furthermore, LR integrations temporally precede both RS and full-site integration when the reactions were performed with pre-spacers capable of catalyzing both transesterification reactions (Figure 4). Additionally, repeat mutations that prevented LR integration also resulted in loss of accurate RS integrations. Moreover, we observed mutations that preserved LR integrations but blocked or altered the site of RS integration but never *vise versa* in both our *in vitro* (Figures 5, 6 and 7) and *in vivo* analyses (Figure 8). Finally, off-target (non-CRISPR) plasmid DNA integrations mapped to a short stretch of sequences that match the LR junction and flanking upstream and downstream nucleotides rather than the RS junction and surrounding sequences, indicating that *S. thermophilus* Cas1–Cas2 integrase exhibits intrinsic sequence recognition of sequences spanning the LR border (Figure 2). A similar preference for integration at the LR vs. RS site was observed *in vitro* for *Streptococcus pyogenes* (7) and *Enterococcus faecalis* (49) type II Cas1–Cas2 integrases. Together, the findings indicate that spacer integration into type II CRISPR repeats normally proceeds with directionality such that the LR junction is initially selected for half-site integration and additional determinants (discussed below) govern the next attack at the RS site that results in a full-site spacer integration at the repeat (Figure 9C).

### First-site integration: Leader-repeat junction

Our results indicate that recognition of a DNA element at the leader-repeat junction, composed of at most 10 bp of the leader and 5 bp of the repeat, is critical for guiding *S. thermophilus* Cas1–Cas2 to make the first step of the two-step, full-site integration reaction at the leader-proximal repeat (Figure 9A). Mutational analyses both *in vivo* (20) and *in vitro* (Figure 1C) demonstrated that 10 bp of the leader proximal to the repeat are necessary and sufficient for directing integration. Moreover, specific block mutations within the repeat immediately downstream of the LR junction disrupted overall efficiency and specific integration at the LR (and RS) junctions while block mutations elsewhere did not prevent accurate LR integrations (Figure 5). As described above, off-target integration events revealed that the *S. thermophilus* Cas1–Cas2 integrase targets sequences that mimic the leader-repeat junction (guanine) which includes ∼ 5 bp of the upstream leader and 5 bp of the downstream repeat (Figure 2C). The recognition of this leader-repeat element by a type II Cas1–Cas2 integrase has been captured by recent X-ray crystallographic structures and revealed base-specific DNA contacts of Cas1 at positions -1 through -4 of the leader as well as +1 and +2 of the repeat (49). The preference for a guanine for the site of integration at both off-target and LR and RS junctions (Figures 2 and 6) appears to be a common determinant for Cas1 proteins of diverse CRISPR systems (7,38,42,49–52,67). In agreement with our findings, other type II-A studies showed that the first 5 bp of the leader sequence specifies sites of integration *in vivo* and that mutations of the leader-proximal repeat sequences affects integration efficiency *in vitro* (7,26,49). Collectively, the results provide strong evidence that type II Cas1–Cas2 proteins have evolved to integrate at the leader-proximal repeat rather than downstream repeats of the CRISPR array via direct recognition of sequences spanning the leader-repeat junction. This contrasts the mechanisms revealed for other (type I) systems that that rely on additional factors such as IHF (integration host factor) that bind at the leader and direct Cas1–Cas2 to integrate at the leader-proximal (first) repeat (28,38,48,53,54).

### Second-site integration: repeat–spacer junction

Once the first step of integration is complete, the remaining 3′-OH terminus of the covalently linked spacer normally performs the second nucleophilic attack precisely at the guanine of the RS border on the opposite strand (Figure 9C and Supplementary Figure S1). Our results suggest that the second site of nucleophilic attack is influenced by multiple factors: (1) it is likely sterically restrained to a relatively narrow range of nucleotides a set distance from the first integration position, (2) it is further specified by a preference for guanine over other bases (Figure 6), (3) it depends upon several determinants within the repeat that likely make contacts with the Cas1–Cas2 integrase (49) to lead to directionality and specificity of the second nucleophilic attack and (4) it is influenced by an element located just upstream of the RS junction (between positions 28–32) that defines integration a fixed distance of 8 bp downstream of the 5′ border of the element both *in vitro* and *in vivo* (Figures 7, 8 and 9C).

Structural studies of a type II Cas1–Cas2 integrase bound to spacer and target DNA during full-site integration (49) suggest that bending of the repeat is necessary for accurate second-site integration (Figure 9C). Moreover, the structural information showed that contacts between the Cas1–Cas2 integrase and the majority of the repeat are mediated by sugar-phosphate backbone interactions rather than base-specific contacts. Second-site recognition appears to be reliant on an accurate first integration step and further guided by multiple determinants distributed throughout the repeat that likely influence repeat bending and positioning of the Cas1 active site at the appropriate guanine residue at the RS border. We noted that the structure showed contact between a non-catalytic Cas1 and repeat residues that correspond to positions 28–29 of the repeat in our experiments. In light of this structural information, it is conceivable that the ruler element that we identified through mutational analysis may represent the breakpoint between the region of the repeat that interacts with the Cas1–Cas2 integrase and the region of the repeat that projects out towards the catalytic Cas1 for second-site integration. The spacing between the non-catalytic Cas1 contact point (with positions 28–29) and the active site of the catalytic Cas1 may correspond to the 8 bp ruler element that we observe for our repeat sequence.

### Type II pre-spacer integration

In summary, we have characterized the *in vitro* properties of *S. thermophilus* Cas1 and Cas2 and found that these two proteins collaborate to catalyze accurate and full-site spacer integration into CRISPR arrays. Our results revealed that type II systems appear to be unique from well-studied type I systems in that the type II Cas1–Cas2 integrases exhibit an intrinsic specificity for LR junctions that drives integration into the leader-proximal repeat instead of downstream repeats and an intrinsic directionality such that the first transesterification reaction is at the LR junction and step two follows at the RS junction. We provide the first evidence supporting a molecular ruler-based mechanism in a type II system that helps guide the second step a fixed distance downstream and functions to maintain the repeat length (Figures 7–9). Such second-site, molecular ruler elements were previously demonstrated to function within type I systems (52,56). Understanding the molecular basis of the ruler-based mechanism that guides the second integration step is an important future goal that will likely require structural and molecular analyses. Future studies are also needed to understand key steps that function upstream of CRISPR spacer integration. For example, there is a gap of knowledge in understanding how viral or plasmid protospacers are recognized and properly processed prior to binding by the Cas1–Cas2 integrase. Furthermore, there is a need for determining the specific roles that Cas9 (29,41) and Csn2 (47,68,69) and perhaps additional host factors play in protospacer to pre-spacer generation and precise PAM removal required for directing spacer integration in a functional orientation in type II CRISPR arrays.

## DATA AVAILABILITY

Sequence data were deposited in the NCBI Sequence Read Archive under the BioProject ID PRJNA548779.

## Supplementary Material

gkz677_Supplemental_File
