# Supplementary material for: CRISPR DNA elements controlling site-specific spacer integration and proper repeat length by a Type II CRISPR–Cas system
Source: Nucleic Acids Res. 2019 Aug 8;47(16):8632–48. doi: 10.1093/nar/gkz677 (PMC6895254; doi:10.1093/nar/gkz677)
Supplement: gkz677_Supplemental_File [file gkz677_supplemental_file.pdf]

Supplementary Material for

CRISPR DNA elements controlling site-specific spacer integration and proper repeat length by a Type II CRISPR-Cas system

Jenny G. Kim, Sandra Garrett, Yunzhou Wei, Brenton R. Graveley and Michael P. Terns

Supplementary Table S1. Plasmids used in this study.

Plasmid name	Plasmid description
pControl	pWAR derived from pWAR228 lacking CRISPR sequence
pCRISPR	pWAR derived from pWAR228 with minimal CRISPR array LRSRS
L1	pCRISPR leader mutation; nucleotides 21 - 32
L2	pCRISPR leader mutation; nucleotides 11 - 20
L3	pCRISPR leader mutation; nucleotides 1 - 10
pCas1/Cas2/Csn2/Cas9+CRISPR	pCas1/Cas2/Csn2/Cas9+minimal CRISPR array
Ins C24	pCas1/Cas2/Csn2/Cas9+CRISPR; insert C, +24
Del A23	pCas1/Cas2/Csn2/Cas9+CRISPR; delete A, +23
Ins C33	pCas1/Cas2/Csn2/Cas9+CRISPR; insert C, +33
Ins CG 33-34	pCas1/Cas2/Csn2/Cas9+CRISPR; insert CG, +33-34
Del C33	pCas1/Cas2/Csn2/Cas9+CRISPR; delete C, +33
Del CA 33-34	pCas1/Cas2/Csn2/Cas9+CRISPR; delete CA, +33-34

Supplementary Table S2. Oligonucleotides used in this study.

Oligos	Sequence (5' – 3')
S4-OS5-F	TCGTTACTGGTGAACCAGTTTCAAT
S4-OS5-R	AACTGGTTCACCAGTAACGACTGAG
WT-F	TTCATTTGAGGTTTTTGTACTCTCAAGATTTAAGTAACTGTACAACTGTTTGACAGCAAATCAAGA
WT-R	TCTTGATTTGCTGTCAAACAGTTGTACAGTTACTTAAATCTTGAGAGTACAAAAACCTCAAATGAA
B1-F	TTCAT**GGTCTTGGGG**TGTACTCTCAAGATTTAAGTAACTGTACAACTGTTTGACAGCAAATCAAGA
B1-R	TCTTGATTTGCTGTCAAACAGTTGTACAGTTACTTAAATCTTGAGAGTACA**CCCCAAGACC**ATGAA
B2-F	TTCATTTGAG**TGGGGGTGCA**TCTCAAGATTTAAGTAACTGTACAACTGTTTGACAGCAAATCAAGA
B2-R	TCTTGATTTGCTGTCAAACAGTTGTACAGTTACTTAAATCTTGAGA**TGCACCCCCA**CTCAAATGAA
B3-F	TTCATTTGAGGTTTTTGTAC**GAGACCTC**TTTAAGTAACTGTACAACTGTTTGACAGCAAATCAAGA
B3-R	TCTTGATTTGCTGTCAAACAGTTGTACAGTTACTTAAA**GAGGTCTC**GTACAAAAACCTCAAATGAA
B4-F	TTCATTTGAGGTTTTTGTACTCTCAAGA**GGGCCTGC**ACTGTACAACTGTTTGACAGCAAATCAAGA
B4-R	TCTTGATTTGCTGTCAAACAGTTGTACAGT**GCAGGCCC**TCTTGAGAGTACAAAAACCTCAAATGAA
B5-F	TTCATTTGAGGTTTTTGTACTCTCAAGATTTAAGTA**CAGTGCACCA**TGTTTGACAGCAAATCAAGA
B5-R	TCTTGATTTGCTGTCAAACA**TGGTGCACTG**TACTTAAATCTTGAGAGTACAAAAACCTCAAATGAA
IR 1-F	TTCATTTGAGGTTT**AACATG**TCTCAAGATTTAAGTAACTGTACAACTGTTTGACAGCAAATCAAGA
IR 1-R	TCTTGATTTGCTGTCAAACAGTTGTACAGTTACTTAAATCTTGAGA**CATGTT**AAACCTCAAATGAA
IR 2-F	TTCATTTGAGGTTTTTGTACTCTCAAGATTTAAGTAACT**CATGTT**CTGTTTGACAGCAAATCAAGA
IR 2-R	TCTTGATTTGCTGTCAAACAG**AACATG**AGTTACTTAAATCTTGAGAGTACAAAAACCTCAAATGAA
IR 3-F	TTCATTTGAGGTTT**AACATG**TCTCAAGATTTAAGTAACT**CATGTT**CTGTTTGACAGCAAATCAAGA
IR 3-R	TCTTGATTTGCTGTCAAACAG**AACATG**AGTTACTTAAATCTTGAGA**CATGTT**AAACCTCAAATGAA
G1C-F	TTCATTTGAG**C**TTTTTGTACTCTCAAGATTTAAGTAACTGTACAACTGTTTGACAGCAAATCAAGA
G1C-R	TCTTGATTTGCTGTCAAACAGTTGTACAGTTACTTAAATCTTGAGAGTACAAAAA**G**CTCAAATGAA
G1A-F	TTCATTTGAG**A**TTTTTGTACTCTCAAGATTTAAGTAACTGTACAACTGTTTGACAGCAAATCAAGA
G1A-R	TCTTGATTTGCTGTCAAACAGTTGTACAGTTACTTAAATCTTGAGAGTACAAAAA**T**CTCAAATGAA
G1T-F	TTCATTTGAG**T**TTTTTGTACTCTCAAGATTTAAGTAACTGTACAACTGTTTGACAGCAAATCAAGA
G1T-R	TCTTGATTTGCTGTCAAACAGTTGTACAGTTACTTAAATCTTGAGAGTACAAAAA**A**CTCAAATGAA
G36C-F	TTCATTTGAGGTTTTTGTACTCTCAAGATTTAAGTAACTGTACAA**G**TGTTTGACAGCAAATCAAGA
G36C-R	TCTTGATTTGCTGTCAAACA**C**TTGTACAGTTACTTAAATCTTGAGAGTACAAAAACCTCAAATGAA
G36A-F	TTCATTTGAGGTTTTTGTACTCTCAAGATTTAAGTAACTGTACAA**T**TGTTTGACAGCAAATCAAGA
G36A-R	TCTTGATTTGCTGTCAAACA**A**TTGTACAGTTACTTAAATCTTGAGAGTACAAAAACCTCAAATGAA
G36T-F	TTCATTTGAGGTTTTTGTACTCTCAAGATTTAAGTAACTGTACAA**A**TGTTTGACAGCAAATCAAGA
G36T-R	TCTTGATTTGCTGTCAAACA**T**TTGTACAGTTACTTAAATCTTGAGAGTACAAAAACCTCAAATGAA
C5-F	TTCATTTGAGGTTT**C**TTGTACTCTCAAGATTTAAGTAACTGTACAACTGTTTGACAGCAAATCAAGA
C5-R	TCTTGATTTGCTGTCAAACAGTTGTACAGTTACTTAAATCTTGAGAGTACAA**G**AAACCTCAAATGAA
C9-F	TTCATTTGAGGTTTTTGT**C**ACTCTCAAGATTTAAGTAACTGTACAACTGTTTGACAGCAAATCAAGA
C9-R	TCTTGATTTGCTGTCAAACAGTTGTACAGTTACTTAAATCTTGAGAGT**G**ACAAAAACCTCAAATGAA
C14-F	TTCATTTGAGGTTTTTGTACTCT**C**CAAGATTTAAGTAACTGTACAACTGTTTGACAGCAAATCAAGA
C14-R	TCTTGATTTGCTGTCAAACAGTTGTACAGTTACTTAAATCTTG**G**AGAGTACAAAAACCTCAAATGAA
C19-F	TTCATTTGAGGTTTTTGTACTCTCAAGA**C**TTTAAGTAACTGTACAACTGTTTGACAGCAAATCAAGA
C19-R	TCTTGATTTGCTGTCAAACAGTTGTACAGTTACTTAAA**G**TCTTGAGAGTACAAAAACCTCAAATGAA

C24-F	TTCATTTGAGGTTTTTGTACTCTCAAGATTTAA**C**GTAACTGTACAACTGTTTGACAGCAAATCAAGA
C24-R	TCTTGATTTGCTGTCAAACAGTTGTACAGTTAC**G**TTAAATCTTGAGAGTACAAAAACCTCAAATGAA
C28-F	TTCATTTGAGGTTTTTGTACTCTCAAGATTTAAGTAA**C**CTGTACAACTGTTTGACAGCAAATCAAGA
C28-R	TCTTGATTTGCTGTCAAACAGTTGTACAG**G**TTACTTAAATCTTGAGAGTACAAAAACCTCAAATGAA
C33-F	TTCATTTGAGGTTTTTGTACTCTCAAGATTTAAGTAACTGTA**C**CAACTGTTTGACAGCAAATCAAGA
C33-R	TCTTGATTTGCTGTCAAACAGTTG**G**TACAGTTACTTAAATCTTGAGAGTACAAAAACCTCAAATGAA
C36-F	TTCATTTGAGGTTTTTGTACTCTCAAGATTTAAGTAACTGTACAA**C**CTGTTTGACAGCAAATCAAGA
C36-R	TCTTGATTTGCTGTCAAACAG**G**TTGTACAGTTACTTAAATCTTGAGAGTACAAAAACCTCAAATGAA
Ins36-F	TTCATTTGAGGTTTTTGTACTCTCAAGATTTAAGTAACTGTACAA**A**CTGTTTGACAGCAAATCAAGA
Ins36-R	TCTTGATTTGCTGTCAAACAG**T**TTGTACAGTTACTTAAATCTTGAGAGTACAAAAACCTCAAATGAA
Ins36-37-F	TTCATTTGAGGTTTTTGTACTCTCAAGATTTAAGTAACTGTACAA**AT**CTGTTTGACAGCAAATCAAGA
Ins36-37-R	TCTTGATTTGCTGTCAAACAG**AT**TTGTACAGTTACTTAAATCTTGAGAGTACAAAAACCTCAAATGAA
Ins36-38-F	TTCATTTGAGGTTTTTGTACTCTCAAGATTTAAGTAACTGTACAA**ATA**CTGTTTGACAGCAAATCAAGA
Ins36-38-R	TCTTGATTTGCTGTCAAACAG**TAT**TTGTACAGTTACTTAAATCTTGAGAGTACAAAAACCTCAAATGAA
Del35-F	TTCATTTGAGGTTTTTGTACTCTCAAGATTTAAGTAACTGTACACTGTTTGACAGCAAATCAAGA
Del35-R	TCTTGATTTGCTGTCAAACAGTGTACAGTTACTTAAATCTTGAGAGTACAAAAACCTCAAATGAA
Del34-35-F	TTCATTTGAGGTTTTTGTACTCTCAAGATTTAAGTAACTGTACCTGTTTGACAGCAAATCAAGA
Del34-35-R	TCTTGATTTGCTGTCAAACAGGTACAGTTACTTAAATCTTGAGAGTACAAAAACCTCAAATGAA
Del33-35-F	TTCATTTGAGGTTTTTGTACTCTCAAGATTTAAGTAACTGTACTGTTTGACAGCAAATCAAGA
Del33-35-R	TCTTGATTTGCTGTCAAACAGTACAGTTACTTAAATCTTGAGAGTACAAAAACCTCAAATGAA
Spacer-Hairpin	TTTCATTTGAGGTTTTTGTACTCTCAAGATTTAAGTAACTGTACAACTGTTTGACAGCAAATCAAGATTCGAATCGATAGATTCGAATCTTGATTTGCTGTCAAACAGTTGTACAGTTACTTAAATCTTGAGAGTACAAAAACCTCAAATGAAA
Leader-Hairpin	AATCTTGATTTGCTGTCAAACAGTTGTACAGTTACTTAAATCTTGAGAGTACAAAAACCTCAAATGAAATTTTGCGATAGCAAAATTTCATTTGAGGTTTTTGTACTCTCAAGATTTAAGTAACTGTACAACTGTTTGACAGCAAATCAAGATT

*underlined annotates mutations

Supplementary Figure S1

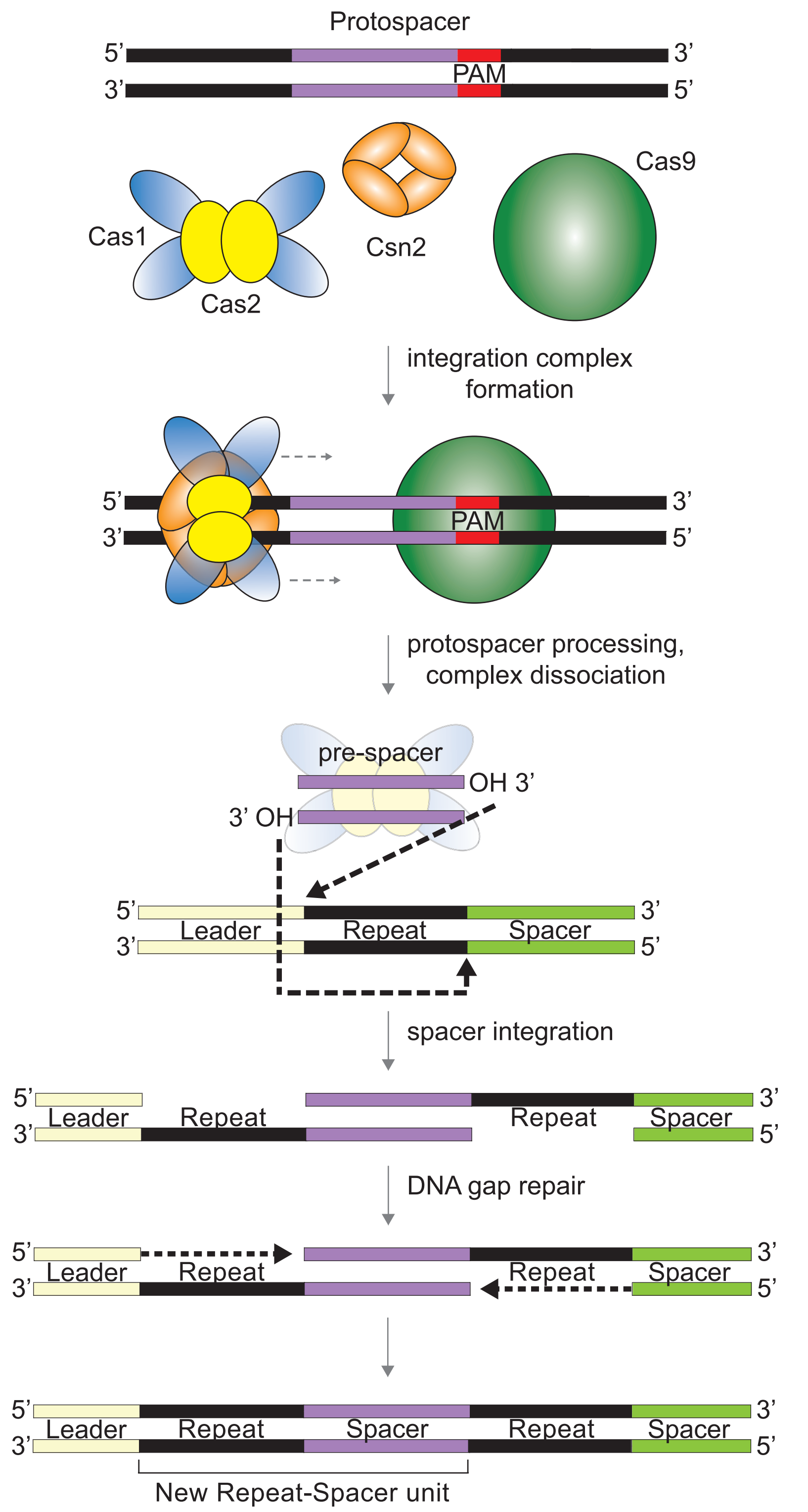

Figure S1. Adaptation model for *S. thermophilus*. CRISPR adaptation involves the capture of foreign invader sequences (protospacers) and generation of pre-spacers in a PAM-dependent manner by the adaptation proteins Cas1, Cas2, Csn2, Cas9 and/or other host factors. Site-directed incorporation of the sequences (pre-spacers) into the host CRISPR locus involves a two-step concerted transesterification reaction by Cas1 and Cas2 in which the 3'-OH groups of the pre-spacer DNA carries out nucleophilic attacks at the 5' ends of the repeat borders. DNA repair events fill in DNA gaps and ensure faithful duplication of a CRISPR repeat.

Supplementary Figure S2

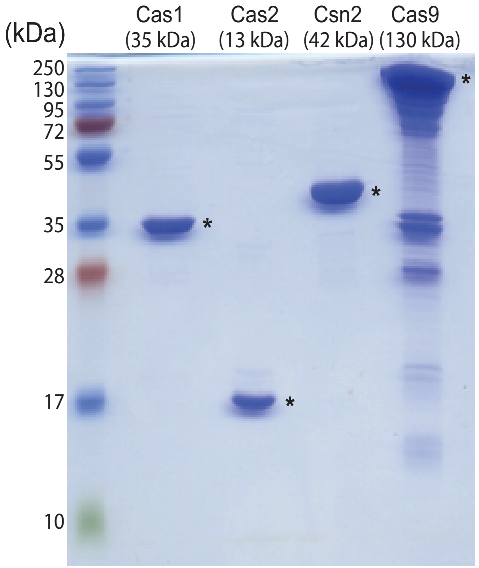

Figure S2. Purified *S. thermophilus* Cas proteins. Purification of individual Cas proteins by Ni^{2+} affinity column chromatography. Products were separated by a SDS-PAGE gel followed by Coomassie blue staining. Bands corresponding to each protein are indicated by a black asterisk while molecular weights of the proteins are listed above.

Supplementary Figure S3

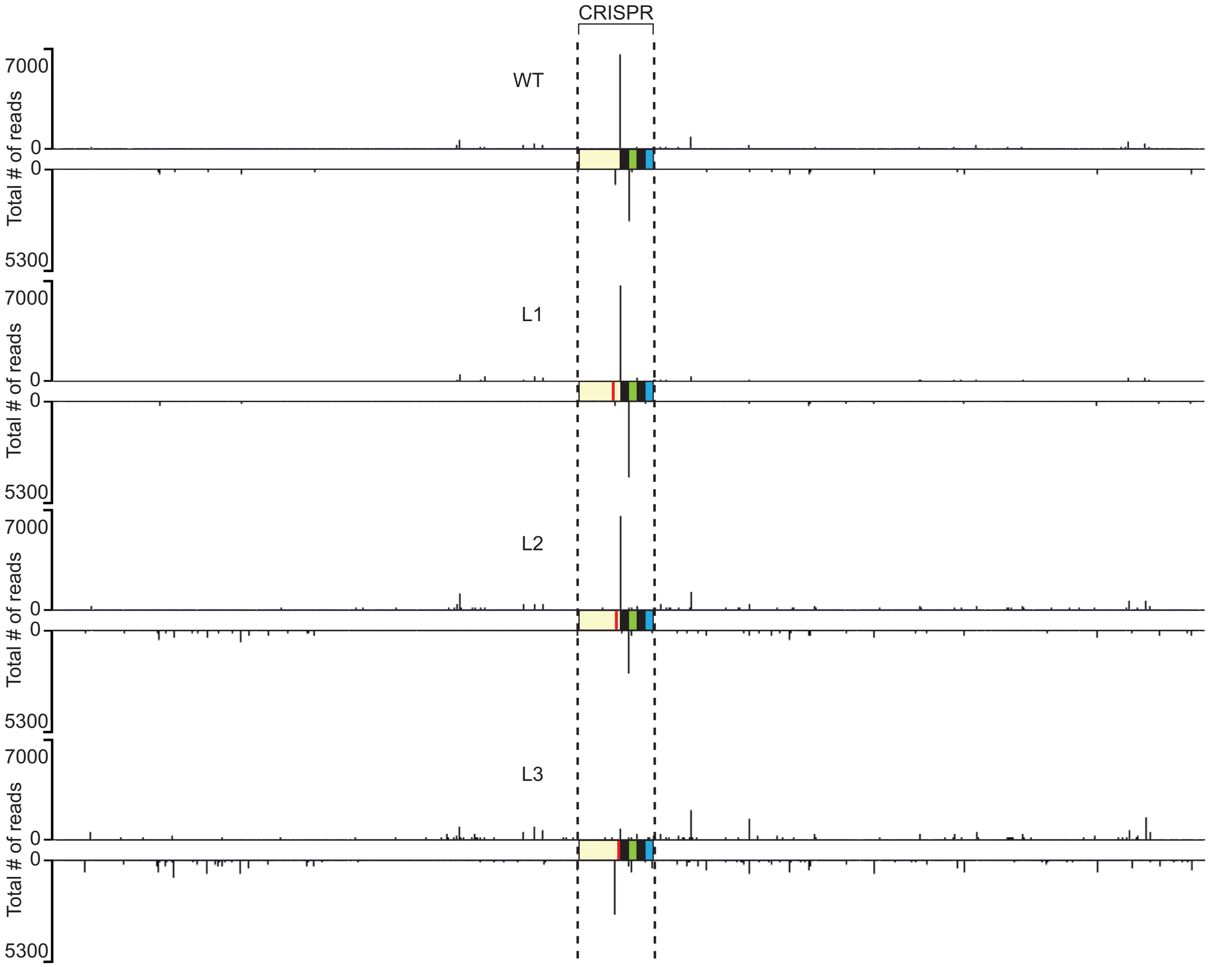

Figure S3. Full plasmid mapping of integration sites on mutated pCRISPR. High-throughput sequence mapping of integrated pre-spacers in mutated pCRISPR plasmids with mutated leader sequences (L1, L2, L3).

Supplementary Figure S4

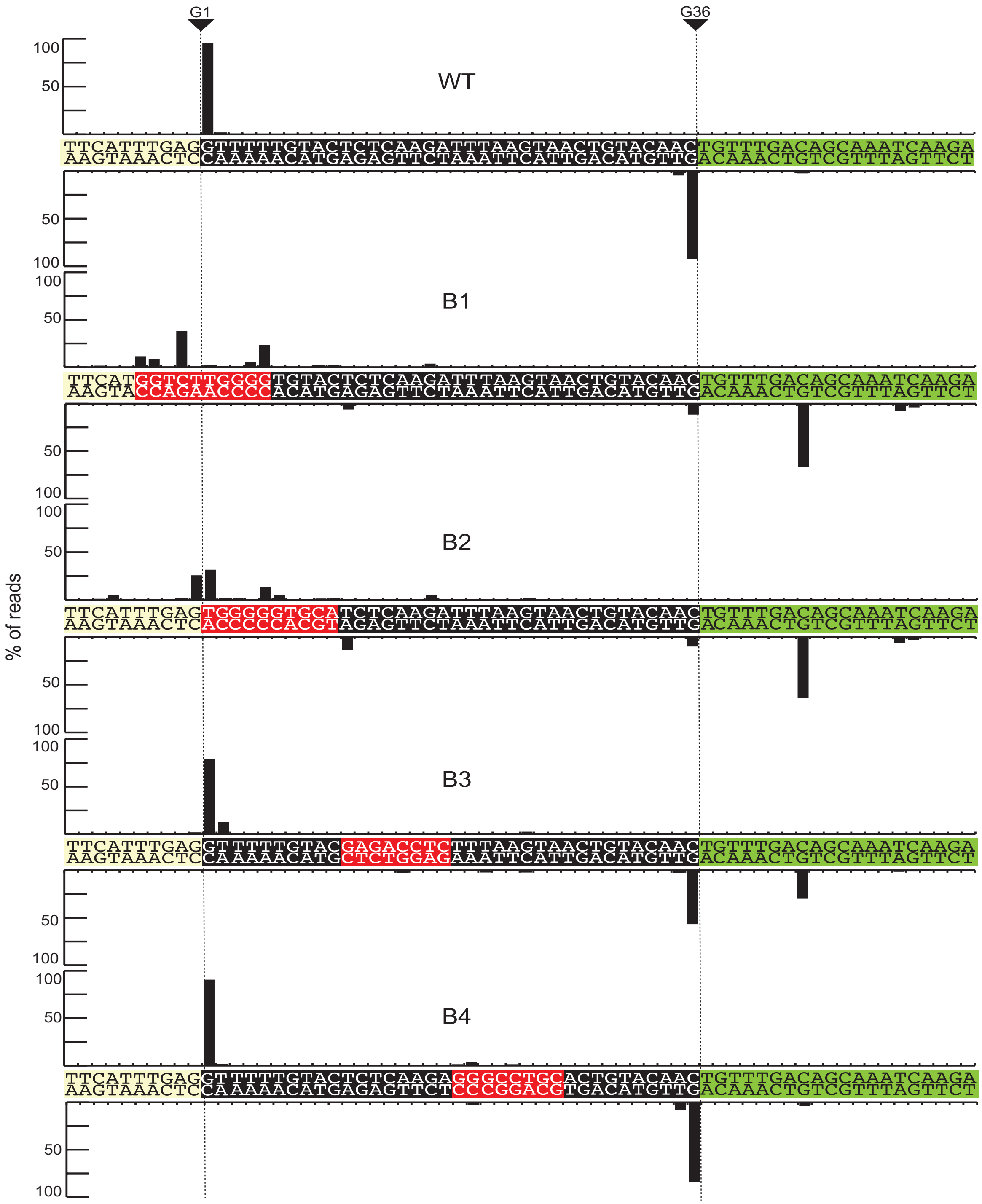

Figure S4. Nucleotide resolution and detailed mapping of integration sites. Histogram plots of high-throughput sequencing analysis of integrated pre-spacers in mutated linear CRISPR targets.

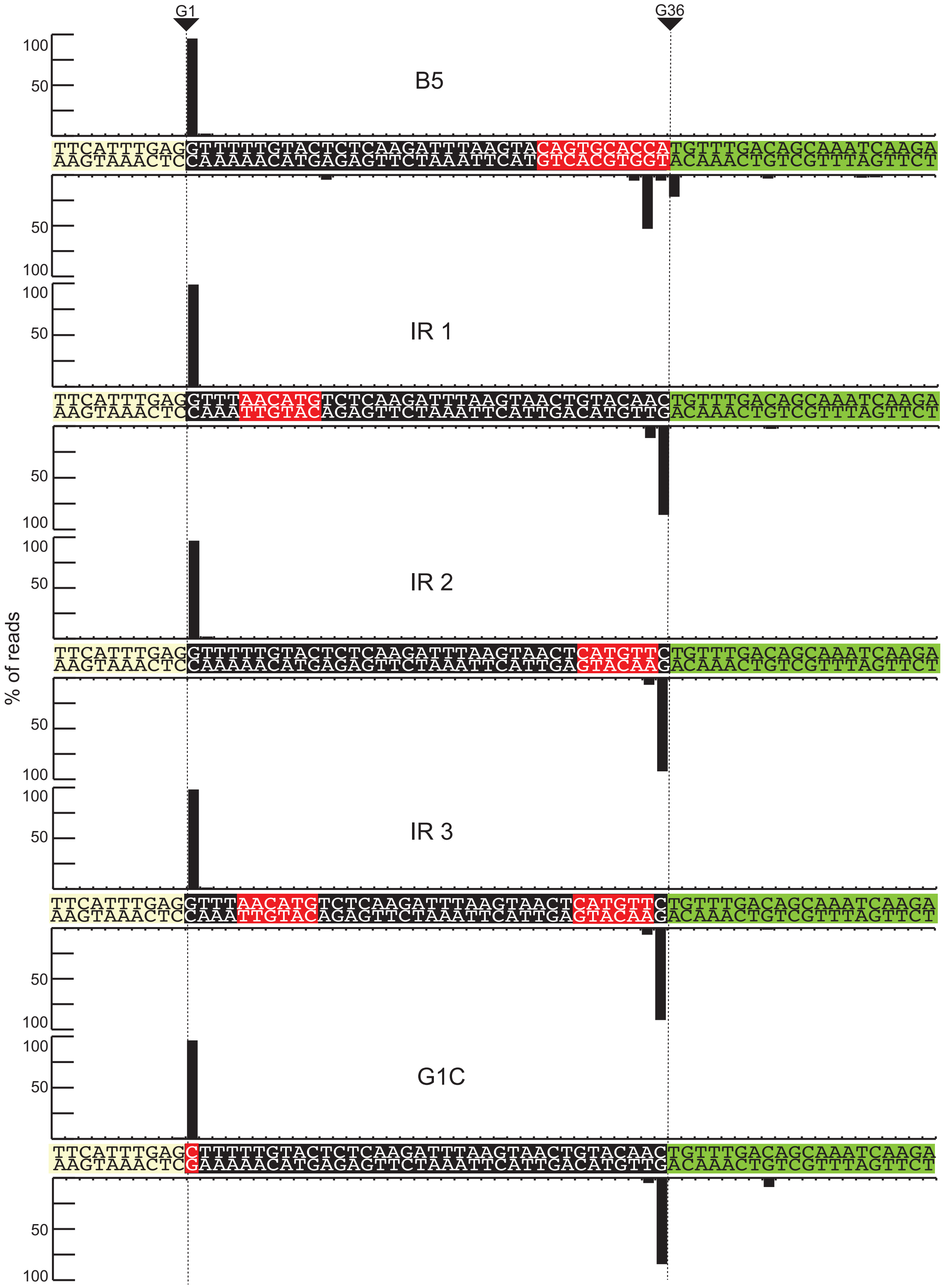

G1
G36
B5
IR 1
IR 2
IR 3
G1C
% of reads
100
50
TTCATTTGAGGTTTTTGTACTCTCAAGATTTAAGTACAGTGCACCATGTTTGACAGCAAATCAAGA
AAGTAAACTCCAAAAACATGAGAGTTCTAAATTCATGTCACGTGGTACAAACTGTCGTTTAGTTCT
TTCATTTGAGGTTTAACATGTCTCAAGATTTAAGTAACTGTACAACTGTTTGACAGCAAATCAAGA
AAGTAAACTCCAAATTGTACAGAGTTCTAAATTCATTGACATGTTGACAAACTGTCGTTTAGTTCT
TTCATTTGAGGTTTTTGTACTCTCAAGATTTAAGTAACTCATGTTCTGTTTGACAGCAAATCAAGA
AAGTAAACTCCAAAAACATGAGAGTTCTAAATTCATTGAGTACAAGACAAACTGTCGTTTAGTTCT
TTCATTTGAGGTTTAACATGTCTCAAGATTTAAGTAACTCATGTTCTGTTTGACAGCAAATCAAGA
AAGTAAACTCCAAATTGTACAGAGTTCTAAATTCATTGAGTACAAGACAAACTGTCGTTTAGTTCT
TTCATTTGAGCTTTTTGTACTCTCAAGATTTAAGTAACTGTACAACTGTTTGACAGCAAATCAAGA
AAGTAAACTCGAAAAACATGAGAGTTCTAAATTCATTGACATGTTGACAAACTGTCGTTTAGTTCT

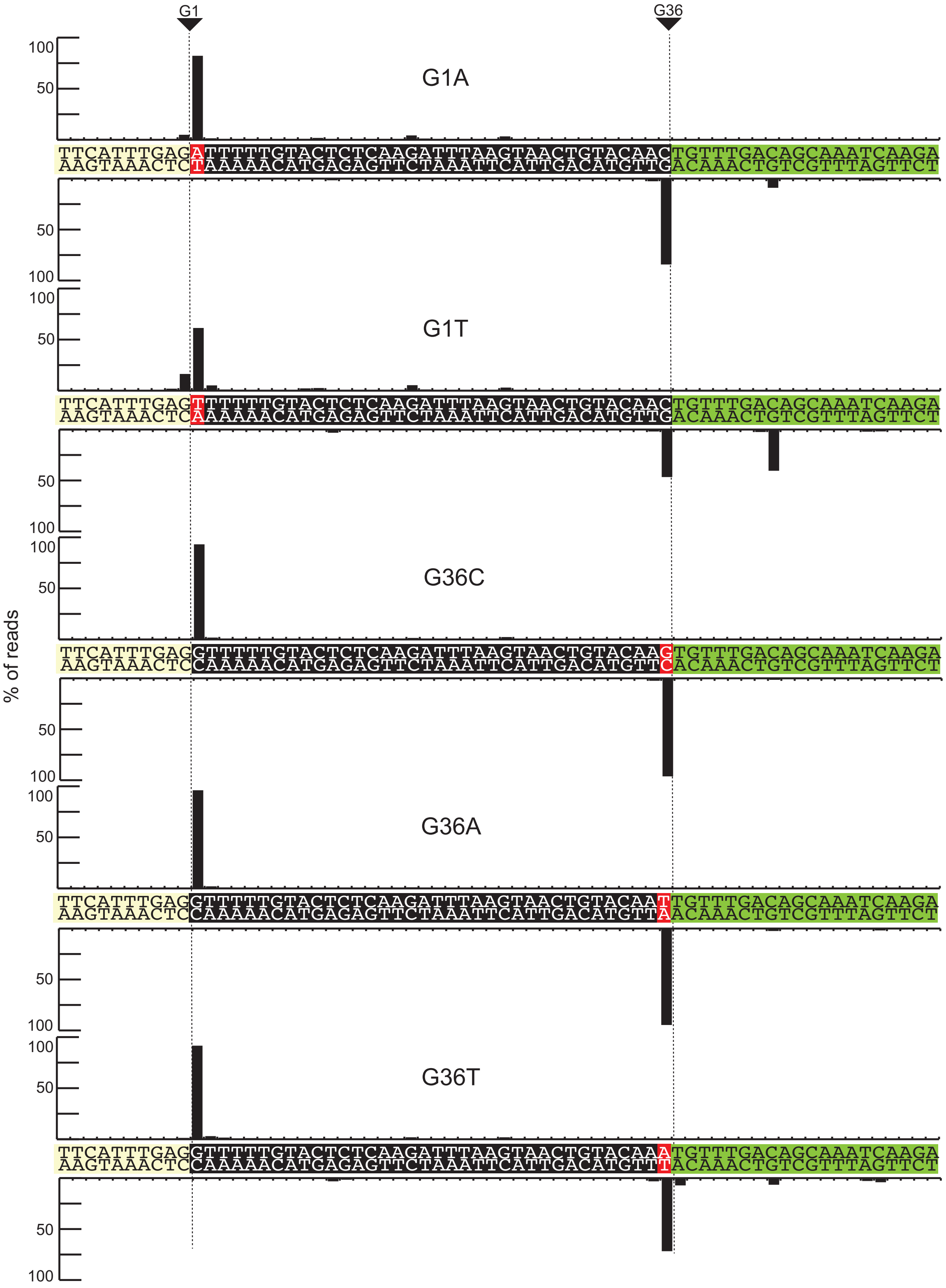
G1
G36
G1A
G1T
G36C
G36A
G36T
% of reads
100
50
TTCATTTGAGATTTTTGTACTCTCAAGATTTAAGTAACTGTACAACTGTTTGACAGCAAATCAAGA
AAGTAAACTCTAAAAACATGAGAGTTCTAAATTCATTGACATGTTGACAAACTGTCGTTTAGTTCT
TTCATTTGAGTTTTTTGTACTCTCAAGATTTAAGTAACTGTACAACTGTTTGACAGCAAATCAAGA
AAGTAAACTCAAAAAACATGAGAGTTCTAAATTCATTGACATGTTGACAAACTGTCGTTTAGTTCT
TTCATTTGAGGTTTTTGTACTCTCAAGATTTAAGTAACTGTACAAGTGTTTGACAGCAAATCAAGA
AAGTAAACTCCAAAAACATGAGAGTTCTAAATTCATTGACATGTTCACAAACTGTCGTTTAGTTCT
TTCATTTGAGGTTTTTGTACTCTCAAGATTTAAGTAACTGTACAATTGTTTGACAGCAAATCAAGA
AAGTAAACTCCAAAAACATGAGAGTTCTAAATTCATTGACATGTTAACAAACTGTCGTTTAGTTCT
TTCATTTGAGGTTTTTGTACTCTCAAGATTTAAGTAACTGTACAAATGTTTGACAGCAAATCAAGA
AAGTAAACTCCAAAAACATGAGAGTTCTAAATTCATTGACATGTTTACAAACTGTCGTTTAGTTCT

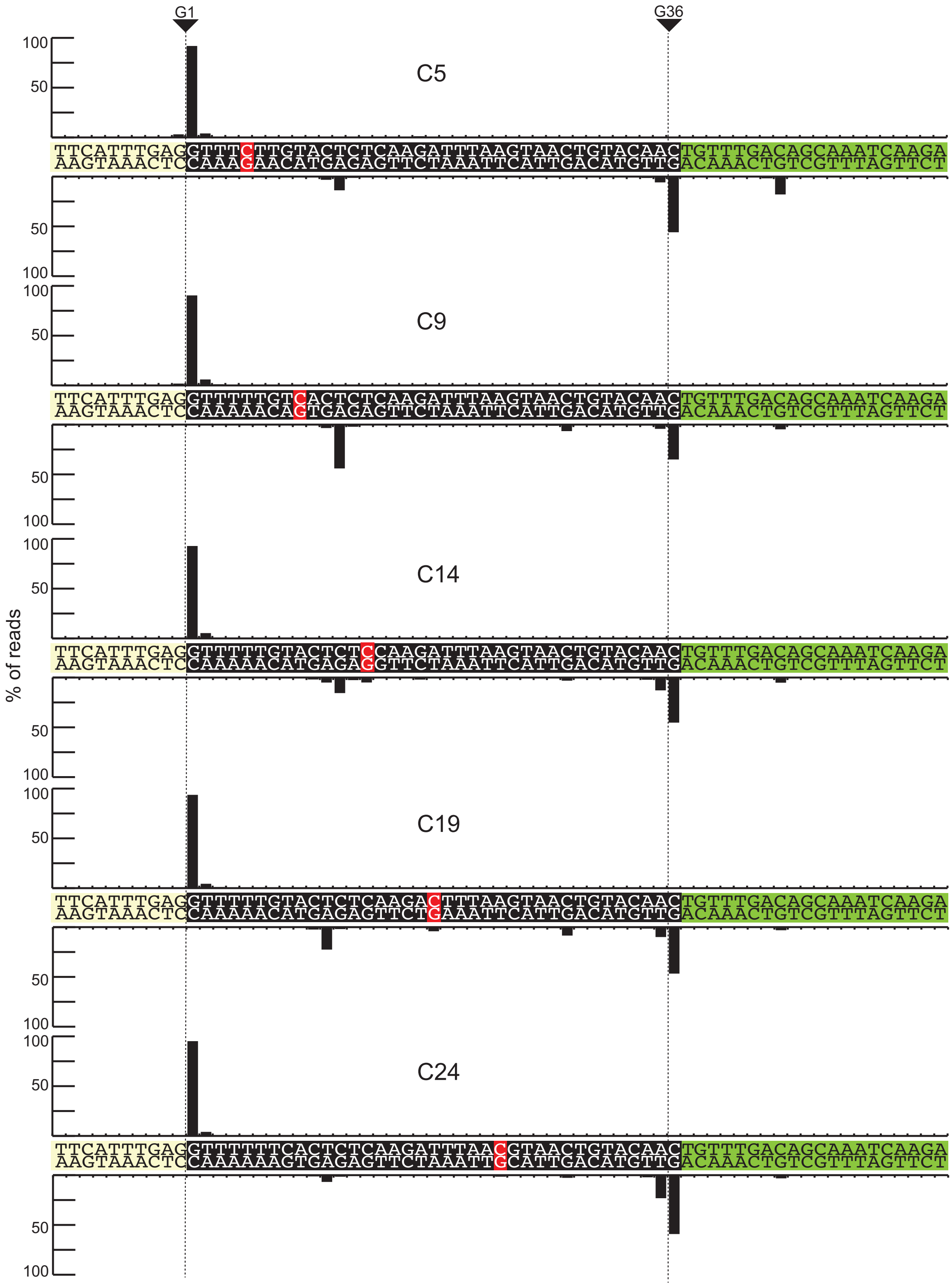

G1
G36
% of reads
100
50
C5
TTCATTTGAGGTTTCTTGTACTCTCAAGATTTAAGTAACTGTACAACTGTTTGACAGCAAATCAAGA
AAGTAAACTCCAAAGAACATGAGAGTTCTAAATTCATTGACATGTTGACAAACTGTCGTTTAGTTCT
C9
TTCATTTGAGGTTTTTGTCACTCTCAAGATTTAAGTAACTGTACAACTGTTTGACAGCAAATCAAGA
AAGTAAACTCCAAAAACAGTGAGAGTTCTAAATTCATTGACATGTTGACAAACTGTCGTTTAGTTCT
C14
TTCATTTGAGGTTTTTGTACTCTCCAAGATTTAAGTAACTGTACAACTGTTTGACAGCAAATCAAGA
AAGTAAACTCCAAAAACATGAGAGGTTCTAAATTCATTGACATGTTGACAAACTGTCGTTTAGTTCT
C19
TTCATTTGAGGTTTTTGTACTCTCAAGACTTTAAGTAACTGTACAACTGTTTGACAGCAAATCAAGA
AAGTAAACTCCAAAAACATGAGAGTTCTGAAATTCATTGACATGTTGACAAACTGTCGTTTAGTTCT
C24
TTCATTTGAGGTTTTTTCACTCTCAAGATTTAACGTAACTGTACAACTGTTTGACAGCAAATCAAGA
AAGTAAACTCCAAAAAAGTGAGAGTTCTAAATTGCATTGACATGTTGACAAACTGTCGTTTAGTTCT

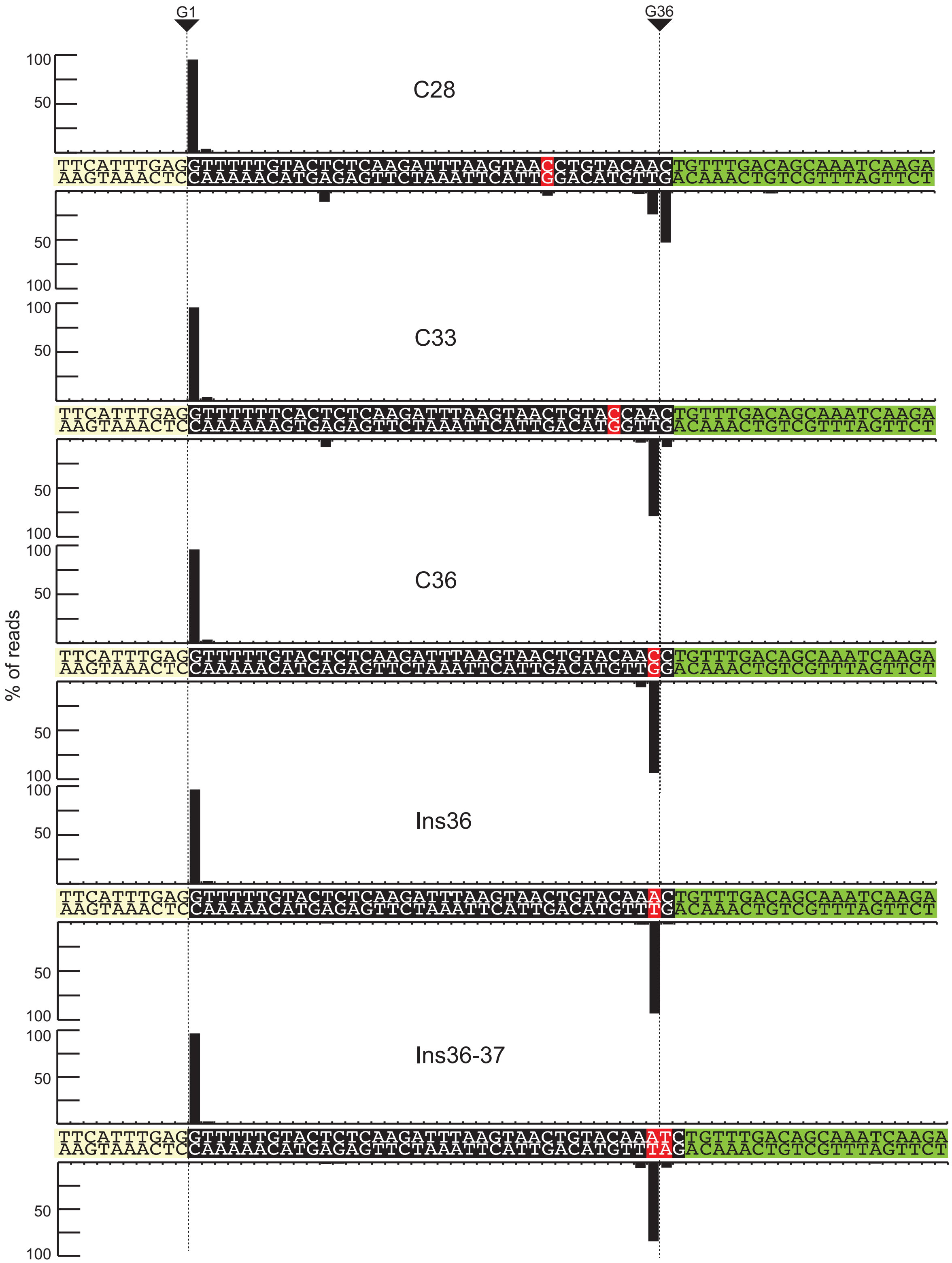
G1
G36
% of reads
100
50
C28
TTCATTTGAGGTTTTTGTACTCTCAAGATTTAAGTAACCTGTACAACTGTTTGACAGCAAATCAAGA
AAGTAAACTCCAAAAACATGAGAGTTCTAAATTCATTGGACATGTTGACAAACTGTCGTTTAGTTCT
C33
TTCATTTGAGGTTTTTTCACTCTCAAGATTTAAGTAACTGTACCAACTGTTTGACAGCAAATCAAGA
AAGTAAACTCCAAAAAAGTGAGAGTTCTAAATTCATTGACATGGTTGACAAACTGTCGTTTAGTTCT
C36
TTCATTTGAGGTTTTTGTACTCTCAAGATTTAAGTAACTGTACAACCTGTTTGACAGCAAATCAAGA
AAGTAAACTCCAAAAACATGAGAGTTCTAAATTCATTGACATGTTGGACAAACTGTCGTTTAGTTCT
Ins36
TTCATTTGAGGTTTTTGTACTCTCAAGATTTAAGTAACTGTACAAACTGTTTGACAGCAAATCAAGA
AAGTAAACTCCAAAAACATGAGAGTTCTAAATTCATTGACATGTTTGACAAACTGTCGTTTAGTTCT
Ins36-37
TTCATTTGAGGTTTTTGTACTCTCAAGATTTAAGTAACTGTACAAATCTGTTTGACAGCAAATCAAGA
AAGTAAACTCCAAAAACATGAGAGTTCTAAATTCATTGACATGTTTAGACAAACTGTCGTTTAGTTCT

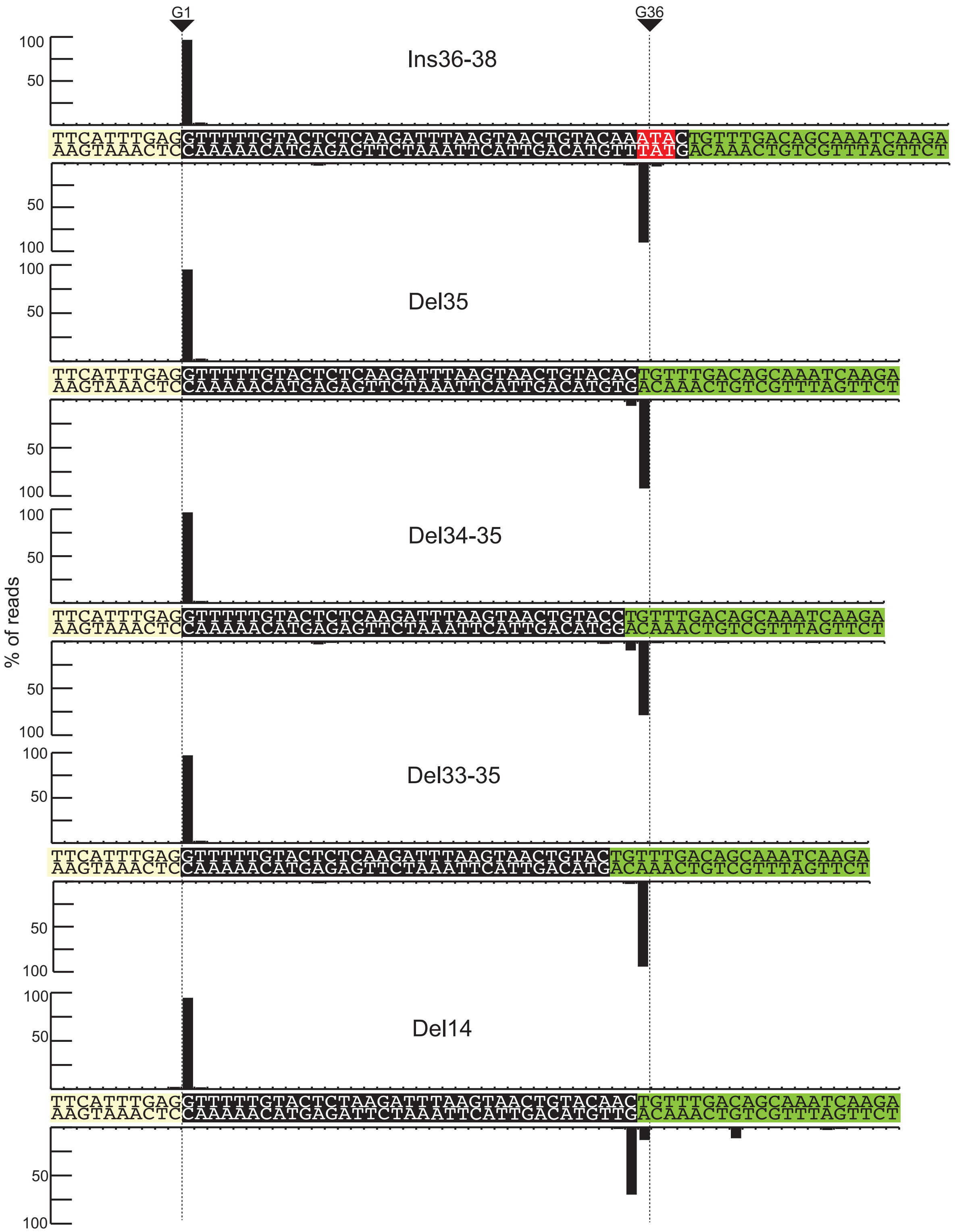

G1
G36
Ins36-38
Del35
Del34-35
Del33-35
Del14
% of reads
100
50
TTCATTTGAGGTTTTTGTACTCTCAAGATTTAAGTAACTGTACAAATACTGTTTGACAGCAAATCAAGA
AAGTAAACTCCAAAAACATGAGAGTTCTAAATTCATTGACATGTTTATGACAAACTGTCGTTTAGTTCT
TTCATTTGAGGTTTTTGTACTCTCAAGATTTAAGTAACTGTACACTGTTTGACAGCAAATCAAGA
AAGTAAACTCCAAAAACATGAGAGTTCTAAATTCATTGACATGTGACAAACTGTCGTTTAGTTCT
TTCATTTGAGGTTTTTGTACTCTCAAGATTTAAGTAACTGTACCTGTTTGACAGCAAATCAAGA
AAGTAAACTCCAAAAACATGAGAGTTCTAAATTCATTGACATGGACAAACTGTCGTTTAGTTCT
TTCATTTGAGGTTTTTGTACTCTCAAGATTTAAGTAACTGTACTGTTTGACAGCAAATCAAGA
AAGTAAACTCCAAAAACATGAGAGTTCTAAATTCATTGACATGACAAACTGTCGTTTAGTTCT
TTCATTTGAGGTTTTTGTACTCTAAGATTTAAGTAACTGTACAACTGTTTGACAGCAAATCAAGA
AAGTAAACTCCAAAAACATGAGATTCTAAATTCATTGACATGTTGACAAACTGTCGTTTAGTTCT

Supplementary Figure S5

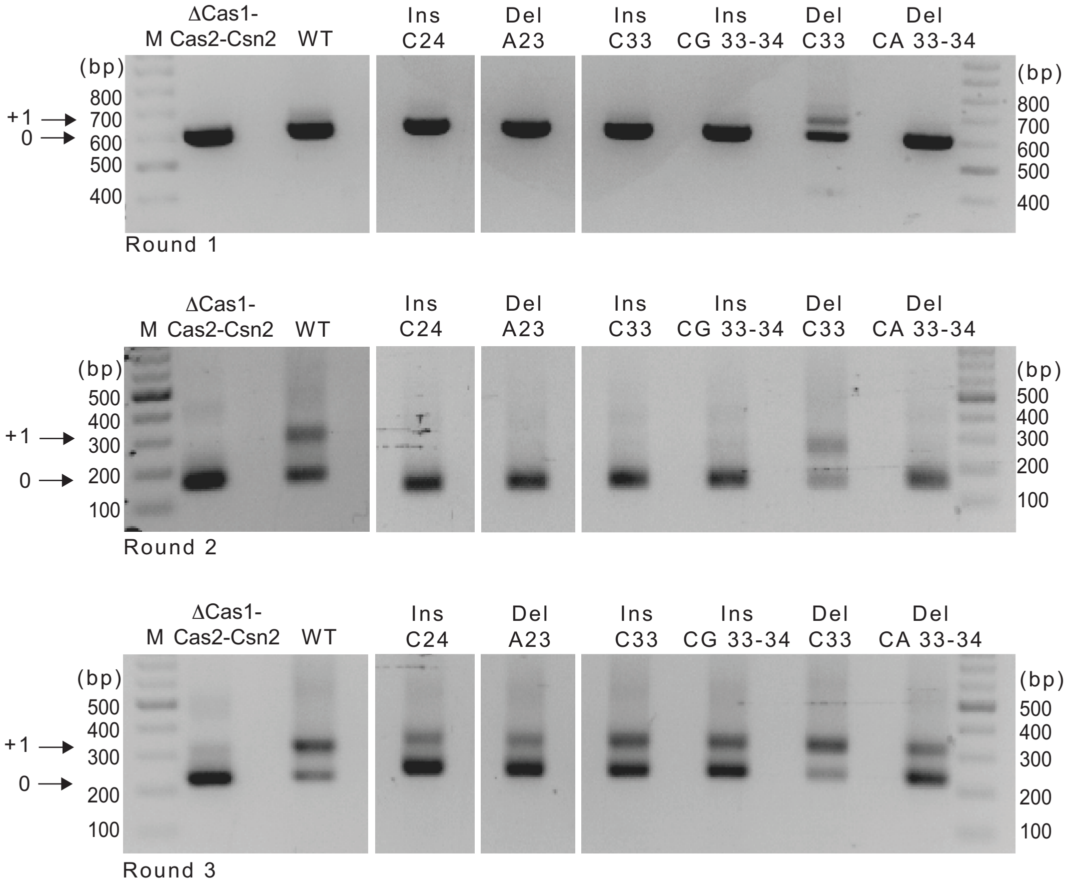

Figure S5. Repeat sequence insertions and deletions reduce adaptation *in vivo*. (Top panel) PCR amplification of the CRISPR array with either wildtype or indicated repeat sequence mutations. Regions in the agarose gel containing expanded CRISPR arrays (+1) were gel excised away from unexpanded CRISPR arrays (0). Extracted DNA was used as template for re-amplification of the array (middle and bottom panels). Adaptation null strain (ΔCas1-Cas2-Csn2) containing the pCRISPR plasmid served as a negative control.

Supplementary Figure S6

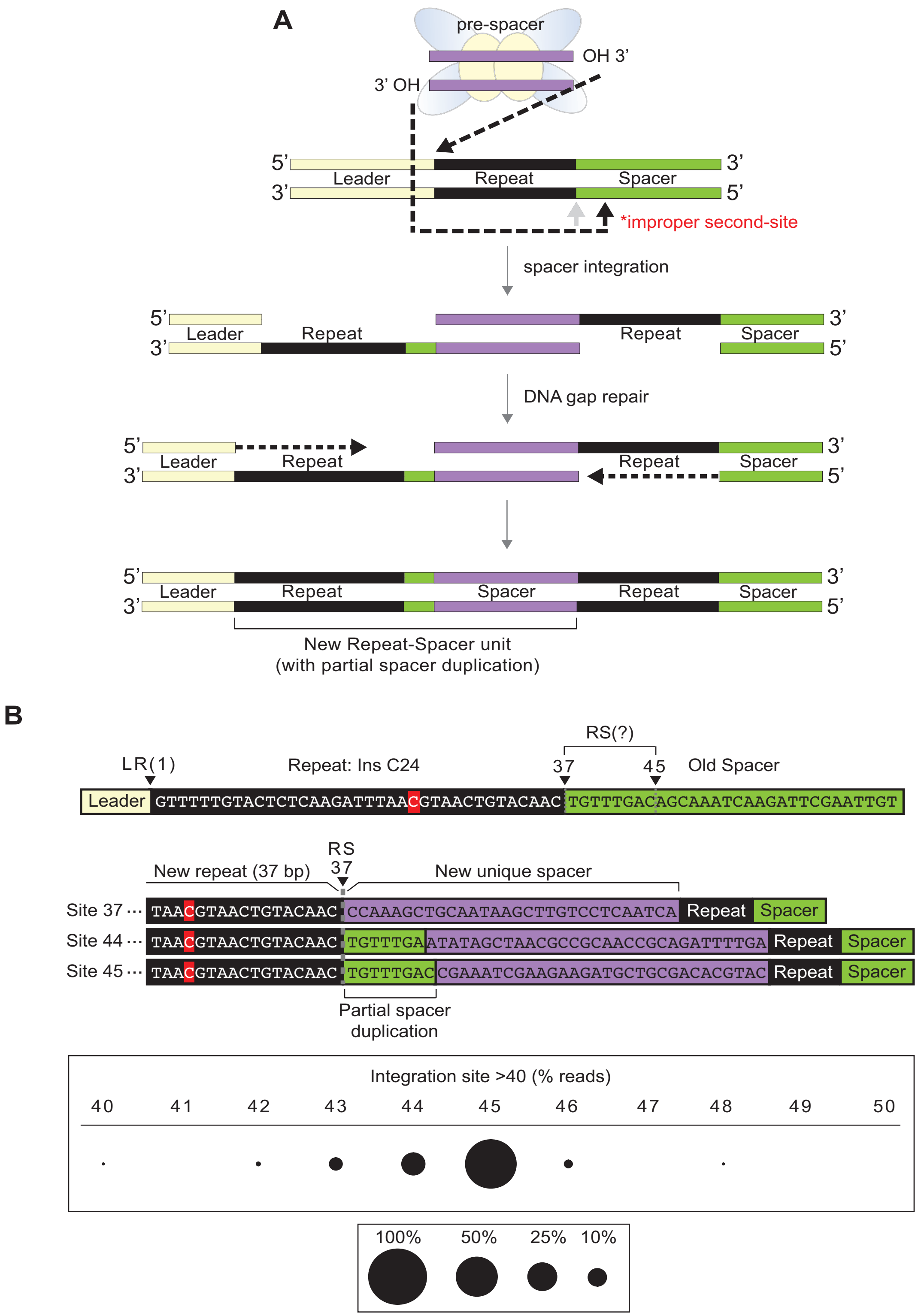

Figure S6. Incorrect second-site integration of the repeat results in partial spacer duplication *in vivo*. (A) Schematic of improper second-site recognition during pre-spacer integration. Misrecognition of the repeat-spacer junction and integration at sites downstream in the adjacent spacer results in partial spacer sequence duplication upstream of the new spacer sequence after DNA gap repair. **(B)** Analysis of "other" sites of integration in Ins C24 repeat mutation strain from Figure 8. Off-target second-site integration events occur predominately at site 44 and 45 within the spacer sequence resulting in partial spacer duplication upstream of the unique spacer of the expanded array.
